# Optimizing SSMMP-Based Fillers with −NH_2_ Functionalization and PIM‑1 Coating for High-Performance CO_2_/CH_4_ and CO_2_/N_2_ Separation in Mixed Matrix Membranes and Thin-Film Composite (TFC) Membrane

**DOI:** 10.1021/acsomega.5c11506

**Published:** 2026-01-22

**Authors:** Henrique Z. Ferrari, Christophe Le Roux, Franciele L. Bernard, Guilherme Dias, Leonardo dos Santos, Pierre Micoud, Stéphane Mazières, François Martin, Sandra Einloft

**Affiliations:** † Post-Graduation Program in Materials Engineering and Technology, 28102 Pontifical Catholic University of Rio Grande Do Sul, Porto Alegre, Rio Grande Do Sul 90619-900, Brazil; ‡ 27091 GET/OMP Laboratory (CNRS, UT3PS, IRD, CNES), Université de Toulouse, ERT Géomatériaux, Toulouse 31400, France; § School of Technology, Pontifical Catholic University of Rio Grande Do Sul (PUCRS), Porto Alegre, Rio Grande Do Sul 90619-900, Brazil; ∥ SOFTMAT Laboratory, CNRS UMR 5623, University of Toulouse, Toulouse Cedex 9 31062, France

## Abstract

Gas separation employing polymeric membranes is limited by the permeability–selectivity trade-off, which has driven the development, among numerous technologies, of mixed matrix membranes (MMMs) that combine highly permeable polymers with fillers capable of enhancing gas selectivity. The compatibility in the filler/polymer interface is therefore essential to design materials with superior separation performances. In this work, MMMs were produced with Pebax-2533, incorporating synthetic silico-metallic mineral particles (SSMMPs) and SSMMP-NH_2_ fillers, both with and without PIM-1 surface coating, and were evaluated in the separation of CO_2_/CH_4_ and CO_2_/N_2_. The membranes were prepared in two types: dense and thin-film composite (TFC). These MMMs were characterized through several techniques, and gas permeation assessments of the dense membranes were conducted at pressures ranging from 1 to 10 bar. The findings demonstrated enhanced thermal, mechanical, and gas separation properties following the addition of the fillers. Specifically, the sample containing 20 wt % SSMMP-NH_2_@PIM-1 achieved a permeability of 501.7 Barrer at 10 bar, representing a 129.8% increase relative to the pure membrane. Additionally, the TFC membrane was fabricated using a self-made porous polysulfone (PSF) support, which was subsequently coated with a selective layer and a protective layer of polydimethylsiloxane (PDMS), achieving a CO_2_ permeance of 575 GPU and selectivities of 12 for CO_2_/CH_4_ and 33 for CO_2_/N_2_. The results demonstrated the beneficial effects of functionalizing the amine groups (−NH_2_) in the fillers, particularly when employing the nonsolvent-induced surface deposition (NISD) technique to coat PIM-1 on the filler surface. The developed materials exhibit promising performance as visualized in the Robeson graph and TFCs target regions, suggesting that they could be suitable for industrial-scale CO_2_ separation with additional development.

## Graphical abstract

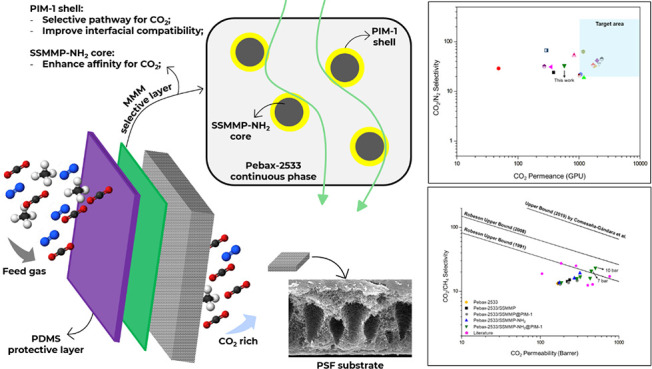

## 1 Introduction

Membrane technology is regarded as an excellent candidate for reducing carbon emissions and supporting the energy transition^1,2^ as it can lower costs and minimize the environmental footprint of the processes in which it is applied,^3^ particularly when compared to other capture and separation technologies.^4^ Polymers are the most widely used materials in membrane gas separation science due to their excellent processability as well as their chemical and mechanical stability.^5,6^ Nevertheless, polymers undergo a trade-off between permeability and selectivity, known as the Robeson Upper Bound,^7,8^ driving the development of membranes with superior performance, which include materials such as the thin-film composite (TFC) membranes, mixed matrix membranes (MMMs),^9–11^ polymers of intrinsic microporosity (PIMs),^12^ and block copolymers of Pebax-1657 and Pebax-2533.^13,14^

Thin-film composite membranes are regarded as the most promising candidates for industrial applications, owing to their thin selective layer (within the nanometer range) that facilitates efficient and rapid gas separation. The selective layer may be constructed from highly permeable polymers or by using mixed matrix membranes (MMMs).^15,16^ MMMs are composite materials created by adding a filler to the polymer matrix, which improves separation capacity by increasing selectivity, permeability, or both.^17^ This happens because the fillers create preferential pathways that facilitate selective permeability or even act as barriers to undesired gases.^18^ Commonly used fillers in MMMs include metal–organic frameworks (MOFs), zeolites, graphene oxide, silica, and others.^19^ In previous work, we applied synthetic silico-metallic mineral particles (SSMMPs) as fillers in MMMs.^20,21^ SSMMPs are obtained from the synthesis procedure described in the literature,^22^ from a magnesium source and a silicon source in a molar ratio of 3:4, respectively.^23,24^ The application of SSMMP as a filler in MMMs is beneficial due to the presence of (−OH) groups available in the −SiOH and −MgOH bonds on the surface, which facilitate the interaction with CO_2_ and the polymer matrix.^25,26^ Furthermore, substitutions in the structure of SSMMPs can be made by inserting new functional groups to improve properties according to the application needs. In this sense, ionic liquids (ILs)^21^ and amino groups are examples of substitutions that occur based on the silicon source.

Despite the intrinsic advantages of MMMs, the compatibility between the filler and the polymer remains a challenge in their production.^27^ Many studies focus on the development of techniques to improve the interface between materials, such as modifying the surface of the filler particles, reducing the particle size,^28^ or inserting new functional groups. Among them, the amino group (−NH_2_) is used because it introduces specific interfacial interactions, including hydrogen bonds and π–π stacking between fillers and matrix.^29^ In addition, (−NH_2_) groups create basic active sites, and when in contact with CO_2_, form Lewis acid–base interactions.^30^ Thus, (−NH_2_) molecules present in the filler structure may improve the sorption and diffusion of the membrane, increasing the selectivity for CO_2_.^30^

Another technique that can be applied in MMMs is the use of core–shell particles, prepared by coating a filler with a polymer, for example.^31,32^ The study by Wu et al.^33^ successfully developed the nonsolvent-induced surface deposition (NISD) technique to prepare MOFs coated with different polymers, including PIM-1 and 6FDA–DAM. Kang et al.^34^ used the same technique to produce core–shell particles based on ZIF-8@PIM-1, to be applied as filler in Pebax-1657 membranes. The results for gas separation demonstrated that the thin polymer layer that coats the filler improved the membrane separation parameters due to the reduction of nonselective voids and improved interfacial adhesion achieved using PIM-1.

Polymers of intrinsic microporosity (PIMs) are macromolecules developed by Budd, McKeown, and colleagues,^12^ which have shown significant progress due to their advantageous properties, including high gas permeability combined with acceptable selectivities.^35–38^ PIMs are synthesized from two components: the first is the structural unit that generates the contortion in the polymer chain, and the second is the linking group that fuses the units, preventing rotation. In the case of PIM-1, the structural unit is the monomer based on spirobisindane (SBI), and the linking group is based on benzodioxin formed during polymerization.^39^ Such contortion sites in the polymer chain generate contorted and super-rigid molecular configurations, causing inefficient packing of the structure and an abundance of free volume.^35,40^ In this way, the interconnected free volume obtained by the lack of rotational freedom results in high gas permeability values.^41^

In this study, SSMMP-based fillers were prepared using two techniques: the first by functionalizing the amino groups (−NH_2_), and the second by surface deposition of PIM-1.^33^ Thus, this work seeks to investigate the effects of polymer surface deposition via nonsolvent-induced surface deposition (NISD) technique on fillers beyond those already studied, extending its application from MOF-based fillers, where its success is already proven.^33,34^ SSMMPs stand out as underexplored fillers in MMMs, offering notable advantages such as rapid, low-cost synthesis. Moreover, SSMMPs possess a highly functional structure rich in reactive groups, enabling tailored modification with CO_2_-philic groups. Furthermore, this study endeavors to elucidate the role of the PIM-1 coating layer in enhancing filler–polymer compatibility, a critical factor for the development of MMMs with improved gas-separation performance. Then, the filler incorporation occurred within the Pebax-2533 polymer matrix, which is characterized by its high permeability to CO_2_.^42^ Pebax-2533 was selected due to its solubility in ethanol, which facilitates the incorporation of fillers coated with PIM-1, since the filler shell is insoluble in the same solvent used for the membrane polymer. Based on the manufactured MMMs, this work strives to verify the effect of the amino group (−NH_2_), the effect of PIM-1 on the surface of the filler, and the synergistic effect between both in the separation of CO_2_/CH_4_ and CO_2_/N_2_ using dense MMMs and TFCs.

## 2 Materials and Methods

### 2.1 Materials

The block copolymer Pebax-2533 (comprising 80 wt % polytetramethylene oxide (PTMO) and 20 wt % polyamide-12­(PA-12)) was provided by Arkema. The monomers 3,3,3′,3′-Tetramethyl-1,1′-spirobiindane-5,5′,6,6′-tetraol (TTSBI, 96%) and 2,3,5,6-Tetrafluoro-1,4-dicyanobenzene (TFTPN, 99%) were purchased from Sigma-Aldrich, and purified before use. Methanol (≥99.9%), dichloromethane (DCM, ≥99.5%), acetone (≥99.5%), *n*-hexane (≥99%), anhydrous potassium carbonate (K_2_CO_3_, ≥99%), *N*,*N*-dimethylformamide (DMF, anhydrous, 99.8%), *N*-methyl-2-pyrrolidone (NMP, anhydrous, 99.5%), chloroform containing amylenes as a stabilizer (≥99%), toluene (anhydrous, 99.8%), tetrahydrofuran (≥99.8%), ethanol (≥99.9%), sodium metasilicate pentahydrate (Na_2_SiO_3_ · 5H_2_O, ≥95%), magnesium acetate tetrahydrate ((CH_3_COO)_2_Mg · 4H_2_O), acetic acid (1M), *N*-[3-(trimethoxysilyl)­propyl]­ethylenediamine (97%), Sylgard 184 PDMS, and polysulfone (PSF) were purchased from Sigma-Aldrich and used without prior purification. N_2_, CH_4_, and CO_2_ gases were purchased from White Martins with purities of 99.99%, 99.99%, and 99.80%, respectively.

### 2.2 PIM-1

#### 2.2.1 Monomer’s Purification

The TTSBI and TFTPN monomers were purified using the method presented by Ameen et al.,^41^ with some adaptations.

The purification of TTSBI was performed by dissolving 15 g of monomer in 400 mL of methanol. A distillation apparatus was employed to slowly remove methanol from the solution. Once the volume of the solution decreased to 100 mL, it was cooled to room temperature, and 300 mL of DCM was added to promote the precipitation of TTSBI. The product was isolated by filtration and dried at 25 °C for 24 h under reduced pressure. After purification, 10.2 g of TTSBI were obtained (68% yield).

TFTPN was purified by dissolving 25 g of monomer in 500 mL of acetone under stirring. Afterward, the insoluble impurities were removed by filtration. Then, to precipitate all of the TFTPN monomer, deionized water was carefully added to the filtered solution. The precipitate was washed 3 times with hexane. Finally, TFTPN was dried under reduced pressure at 25 °C for 24 h, and 20.9 g (84% yield) of pure TFTPN were isolated.

#### 2.2.2 Synthesis of PIM-1

The polymer PIM-1 was produced by the reaction between TFTPN and TTSBI monomers at high-temperature conditions, using a method described in other works.^41,43–45^ TFTPN monomer (4.002 g, 20 mmol), TTSBI monomer (6.808 g, 20 mmol), and potassium carbonate K_2_CO_3_ (8.292 g, 60 mmol) were introduced in a 250 mL round-bottom three-necked flask equipped with a condenser. The reaction mixture was put under reduced pressure for 30 min. The flask was then filled with argon, and 36 mL of NMP and 12 mL of dry toluene were then added. The solution formed was heated to 160 °C under stirring and an inert atmosphere. After 40 min, methanol was added to quench the PIM-1 formation reaction.

The synthesized PIM-1 polymer was recovered by filtration on a glass filter and promptly dissolved completely in chloroform (a concentration of 1 g/20 mL was used). Then, PIM-1 was slowly dropped into a beaker filled with 400 mL of methanol. The precipitated polymer was collected by a glass filter. A new dissolution in chloroform, followed by reprecipitation in methanol, as previously described, was performed. Finally, PIM-1 was washed with an excess of methanol and dried at 100 °C for 48 h (84% yield). PIM-1 was characterized by SEC, NMR, and FTIR (see Section 3.1).

### 2.3 Filler Synthesis

#### 2.3.1 Synthesis of SSMMP and SSMMP-NH_2_

SSMMP are synthesized from a suitable Mg/Si ratio (3/4)^25^ using Na_2_SiO_3_ · 5H_2_O as the Si source and (CH_3_COO)_2_Mg · 4H_2_O as the Mg source. The synthesis procedure is the same as that for the sample SSMMP-M2 of ref. 25. Pure SSMMP was produced from the dissolution of 5.300 g (25 mmol) of Na_2_SiO_3_ · 5H_2_O in milli-Q water and the dissolution of 4.013 g (18.75 mmol) of (CH_3_COO)_2_Mg · 4H_2_O in milli-Q water with the addition of 1N acetic acid, followed by mixing the two solutions under constant stirring to obtain a precipitate.

To produce SSMMP with amino groups (−NH_2_), patent application WO2013093339 ^22^ was used, with 20% of the silicon source replaced by *N*-[3-(Trimethoxysilyl)­propyl]­ethylenediamine. For this purpose, three solutions were prepared: Solution 1) Na_2_SiO_3_ · 5H_2_O (16.971 g, 80 mmol) was dissolved in milli-Q water. Solution 2) *N*-[3-(Trimethoxysilyl)­propyl]­ethylenediamine (4.447 g, 20 mmol) was added to milli-Q water. Solution 3) (CH_3_COO)_2_Mg · 4H_2_O (16.084 g, 75 mmol) was dissolved in milli-Q water together with a 1N acetic acid solution. Then, under mechanical stirring, solution 2 was added to solution 1, and then solution 3 was immediately added to this prepared solution (1 + 2). As a result, a white precipitate was immediately formed.

Purification of both synthesized materials was performed by centrifugation and washing with milli-Q water (3 times), followed by drying in an oven at 70 °C for 24 h.

#### 2.3.2 Synthesis of SSMMP@PIM-1 and SSMMP-NH_2_@PIM-1 Particles

The core–shell fillers, based on SSMMP and SSMMP-NH_2_ coated with PIM-1, were synthesized as presented in the procedure available in Kang et al.^34^ For that, 0.5 g of SSMMP and SSMMP-NH_2_ were respectively stirred and mixed with 0.05 g of PIM-1 in 20 mL of dichloromethane. Afterward, the precipitation of the particles was carried out by adding 40 mL of petroleum ether under strong stirring. The material was separated by centrifugation at 12,000 rpm for 15 min, succeeded by washing with petroleum ether. Afterward, the materials were dried at 70 °C for 24 h. The fillers were named as SSMMP@PIM-1 and SSMMP-NH2@PIM-1, and as a characteristic of the synthesis, the color of the fillers after superficial deposition of PIM-1 was changed from white to yellow.

### 2.4 Membrane Preparation

MMMs based on Pebax-2533 were produced in accordance with the literature,^46,47^ using solution casting followed by solvent evaporation. Specific amounts of fillers were dispersed in ethanol. Then Pebax-2533 was added in 2 steps (priming method)^48^ (Table 1 and Figure S1) under stirring at 70 °C for 6 h to produce an 8 wt % polymer/solvent solution. Afterward, the polymer solution was cooled naturally to ambient temperature under stirring and sonicated for 20 min. Then, the solution was immediately poured into a Petri dish on a level surface and covered with another dish to delay solvent evaporation. After 48 h, the MMM was dried in a vacuum oven at 60 °C for 24 h. For the pure membrane, 8 wt % of Pebax-2533 was produced without incorporating any filler and using the same drying procedure.

**Table 1:** Experimental Methodology and Quantities for Preparing MMMs

Membrane	Filler (g)	Total Polymer (g)	Polymer First Stage (g)	Polymer Second Stage (g)	Polymer/Solvent (wt %)
Pebax-2533	-	1.0	1.0	-	8
MMM with 0.5 wt %^a^	0.005	0.995	0.0995	0.8955	8
MMM with 10 wt %^a^	0.10	0.90	0.09	0.81	8
MMM with 20 wt %^a^	0.20	0.80	0.08	0.72	8

a Quantities to produce all MMMs, which include: Pebax-2533/SSMMP, Pebax-2533/SSMMP@PIM-1, Pebax-2533/SSMMP-NH_2_, and Pebax-2533/SSMMP-NH_2_@PIM-1.

Membrane samples were designated as Pebax-2533, Pebax-2533/SSMMP, Pebax-2533/SSMMP@PIM-1, Pebax-2533/SSMMP-NH_2_, and Pebax-2533/SSMMP-NH_2_@PIM-1 followed by the filler wt %.

### 2.5 Synthesis of TFC Membranes

#### 2.5.1 Production of Porous Polysulfone (PSF) Support

The porous polysulfone support was produced with the methodology presented by Jiang et al.,^49^ with minor modifications. Polysulfone was first dried at 90 °C for 24 h in an oven before use to remove moisture. It was then dissolved in anhydrous dimethylformamide (DMF) under strong stirring at 70 °C for 24 h at a polymer/solvent concentration of 25 wt %. Next, the solution was degassed with a vacuum pump to ensure a bubble-free film. The automatic film applicator was used to prepare the polymeric film. For this, the solution was placed between the glass and the 300 μm casting knife, which was operated at a speed of 30 mm/s. The glass with the freshly molded solution was promptly immersed in a coagulation bath with deionized water to induce phase inversion. Every 12 h, the deionized water was changed, and the film was immersed again to remove the residual DMF. The water renewal procedure was carried out 5 times.

Finally, to prevent the collapse of the porous structure of the PSF membrane used as a support material for TFC, the solvent was changed. The PSF support was removed from the deionized water and instantly placed in a methanol bath for 30 min, followed by another immersion in hexane for 30 min. The PSF support was dried under vacuum at 60 °C for 24 h. As a result, supports measuring 15 x 20 cm (width x length) were obtained.

#### 2.5.2 Production of TFC-MMM

The TFC-MMMs were produced based on the methodology presented by Kang et al.^50^ and Min et al.,^51^ with minor modifications in solution concentration for the selective and protective layers and for the equipment. In this study, a BGD 219 automatic film applicator was used. Initially, solutions for forming selective and protective layers were prepared. In this work, the layer that promotes separation was composed of the best polymer/filler combination observed from the gas separation results of the dense MMMs. Consequently, a diluted solution of 3 wt % polymer/solvent was prepared, adhering to the same experimental procedure as the dense membranes. In this case, it was followed by the addition of 20 wt % of filler relative to the polymer, and then Pebax-2533 was added while stirring at 70 °C for 6 h. The PDMS solution used as a protective layer was produced by dissolving in hexane with a concentration of 2 wt %.

To produce TFC-MMM, the prepared porous PSF support was cut into rectangles measuring 5 cm × 14 cm (width × length) and adhered to the glass. Then, the polymer solution was applied to the PSF support by using the automatic film applicator. The material was dried for 12 h at room temperature, succeeded by drying at 60 °C for 24 h. Next, PDMS solution was added onto the previously prepared selective layer using the automatic film applicator, and to cross-link the PDMS, the TFC was dried at 60 °C for 12 h. In this work, applying PDMS over the selective layer was done to correct defects and thus improve selectivity.^49,52^

### 2.6 Characterization

Weight-average molar mass (*M*_w_), number-average molar mass (*M*_n_), and dispersity (*Đ*) of synthesized PIM-1 were measured by ultrahigh performance liquid chromatography-size exclusion chromatography (UHPLC-SEC) (SOFTMAT laboratory, Toulouse, France). Three samples were prepared at a concentration of 1 mg mL^–1^ of PIM-1 in THF and examined at a flow rate of 0.5 mL/min, with a 10 μL injection, at 30 °C using two columns, Waters APC-XT 125 + 200_150 mm. The analysis was performed in duplicate, and the results were evaluated in the ASTRA 8 software. For PIM-1, the specific refractive index increment (d*n*/d*c*) was 0.2381, according to the literature.^53^

Bruker Avance III HD 400 spectrometer equipped with a 4 mm probehead was used for solid-state NMR experiments (LCC laboratory, Toulouse, France). The sample was spun at 9 kHz at 298 K. ^13^C–CP/MAS spectra were recorded with a recycle delay of 1.5 s and a contact time of 2 ms. Chemical shifts for ^13^C are relative to those of TMS.

The surface area of the fillers was measured in triplicate by the Brunauer–Emmett–Teller (BET) method (LAMAT laboratory, Porto Alegre, Brazil). N_2_ isotherms for adsorption and desorption were recorded at 77 K using the TriStar II Plus.

Variations in glass transition temperature (*T*_g_) of the membranes based on Pebax-2533 after the incorporation of fillers were measured by differential scanning calorimetry (DSC), performed using TA Instrument Q20, with heating from −80 to 250 °C, with a ramp of 10 °C/min under an inert atmosphere (LOR laboratory, Porto Alegre, Brazil).

Thermogravimetric analysis (TGA) was conducted on all produced samples (membranes and fillers) using TA Instruments SDTQ600 equipment (LOR laboratory, Porto Alegre, Brazil). The sample was heated to 700 °C at 10 °C min^–1^ under a nitrogen atmosphere.

The surface morphology, cross-section images of polymeric membranes, and the structure of the fillers were verified by scanning electron microscopy (SEM) using a Philips model XL 30 equipment (LABCEMM laboratory, Porto Alegre, Brazil). For the cross-sections, cryogenic fracturing was performed with liquid nitrogen. The filler particle size was determined from SEM images using ImageJ 1.53m software by analyzing a minimum set of 30 particles per sample.^54^

The structure of the fillers was also evaluated by transmission electron microscopy (TEM) using Model Tecnai G2 T20 FEI equipment (LABCEMM laboratory, Porto Alegre, Brazil).

Fourier transform infrared spectroscopy (FTIR) using a PerkinElmer Frontier spectrometer was used to verify the functional groups of pure and modified fillers (LOR laboratory, Porto Alegre, Brazil).

XRD patterns for powders were analyzed between the 2–80° 2θ range using a Bruker D8 Advance A25 equipment (GET laboratory, Toulouse, France).

The water contact angle of the different surfaces of the TFC membrane was measured in triplicate by a Phoenix 300 goniometer (LOR laboratory, Porto Alegre, Brazil).

The produced membranes underwent dynamic mechanical analysis (DMA) using TA Instruments Model Q800 equipment (LOR laboratory, Porto Alegre, Brazil). Tension/strain tests were conducted in triplicate with rectangular polymer films.

### 2.7 Gas Permeation Measurements

The membranes were subjected to permeability tests to pure gases CO_2_, CH_4_, and N_2_ in a constant volume system with a pressure transducer that computes pressure versus time (d*p*/d*t*) downstream of the sample. The tests were performed at 1, 4, 7, and 10 bar, at 25 °C, and in triplicate. Other system specifications can be found in previous work.^55^ Eqs 1 and 2 were used to assess the gas permeability and the membrane’s ideal selectivity, respectively.^56^

1 $$P=J_{i}\frac{l}{\Delta p}=\frac{273.15Vl}{76A\Delta pT}\left(\frac{dp}{dt}\right)$$

2 $$\alpha_{i/j}=\frac{P_{i}}{P_{j}}$$

where *J_i_* is the gas flux, *T* represents the test temperature (K), *A* represents the area (cm^2^), *V* represents the volume of the permeation equipment (cm^3^), *p* is the experiment pressure, *l* is the thickness of the membrane (cm), *P* is the permeability in cm^3^ (STP)­cm/(cm^2^ s cmHg) [1 Barrer = 1 × 10^–10^ cm^3^(STP)­cm cm^–2^ s^–1^ cmHg^–1^] for dense membranes and permeance in GPU [10^–6^ cm^3^(STP)­cm^–2^ s^–1^ cmHg^–1^] for TFC membranes, and α_*i*/_ *_j_* is the ideal selectivity.

The coefficients for the solution-diffusion mechanism^57^ (eq 3) were also evaluated to understand the role of fillers in the dense polymer matrix and also the effect of PIM-1 coating on the SSMMP and SSMMP-NH_2_ fillers. For diffusion coefficient determination, the time-lag method^58^ was used (eq 4).

3 $$S=\frac{P}{D}$$

4 $$D=\frac{l^{2}}{6\theta }$$

*S* is the solubility coefficient (cm^3^ (STP)/(cm^3^ cmHg)), *D* is the diffusion coefficient (cm^2^/s), θ is the time-lag (s), and *l* is the membrane thickness (cm).

## 3 Results

### 3.1 Synthesis and Characterization of PIM-1

The synthesized PIM-1 was characterized by SEC, NMR, and FTIR. SEC analysis of PIM-1 showed *M*_n_ = 83.5 kg mol^–1^, *M*_w_ = 123 kg mol^–1^, and dispersity (*Đ*) = 1.47, in agreement with previously described in the literature.^59,60^ Additionally, these results are consistent with the values reported by Du et al.^61^ for PIM-1 synthesized via the high-temperature polymerization method, in which a high molecular weight polymer was obtained in a shorter time than the low-temperature method, exhibiting similar polydispersity indices characteristic of polycondensation reactions.

PIM-1 ^13^C-MAS NMR spectra are presented in Figure 1 and show values δ ppm 148.8, 139.5, 111.0, 94.4, 57.5, 43.2, and 30.0, in agreement with previous works available in the literature^62–65^ proving the formation of the PIM-1.

**Figure 1:**
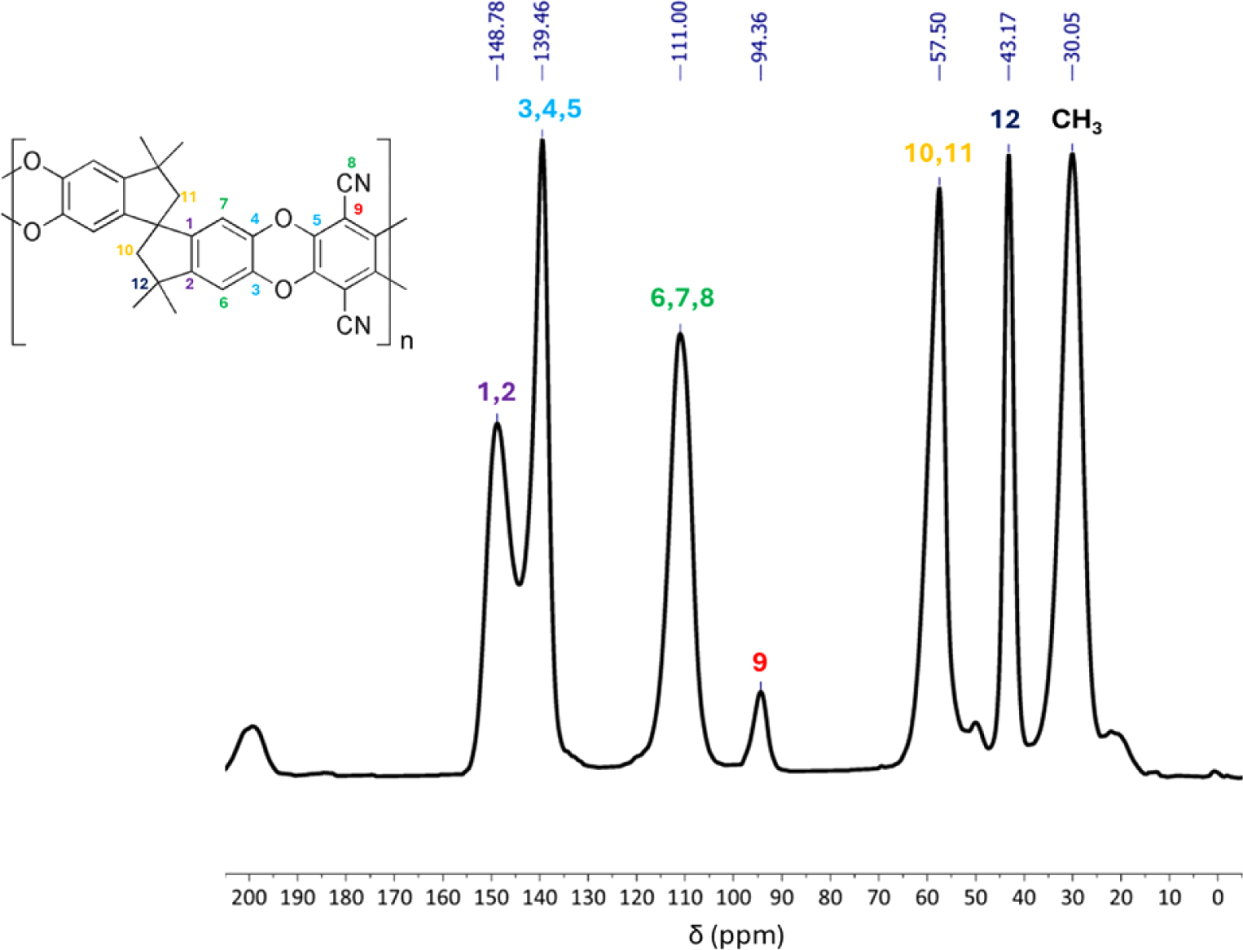
^13^C-MAS NMR spectra of PIM-1.

FTIR spectra of PIM-1 are shown in Figure S2. The stretching of the C–H bond can be observed through the characteristic bands at 2950, 2930, and 2860 cm^–1^. The band at 2239 cm^–1^ is associated with the nitrile groups (C≡N stretching), the band at 1604 cm^–1^ is related to the stretching vibrations of aromatic C=C, and the characteristic band at 1009 cm^–1^ corresponds to the C–N stretching. The region between 1320 and 1250 cm^–1^ is attributed to the C–O stretching mode.^43,66,67^

### 3.2 Characterization of Fillers

Figure 2 displays the SEM images of the synthesized fillers, while the images in Figure S3 illustrate the characteristics observed without the aid of instruments. SSMMP, SSMMP-NH_2_, SSMMP@PIM-1, and SSMMP-NH_2_@PIM-1 exhibit the morphology of nanometer-sized spherical particle clusters. On average, the particles maintain similar sizes, and after the modifications, no significant differences in the morphology of the samples were noted. Furthermore, SSMMP-based particles have average sizes of 30 to 50 nm, similar to other studies.^21^

**Figure 2:**
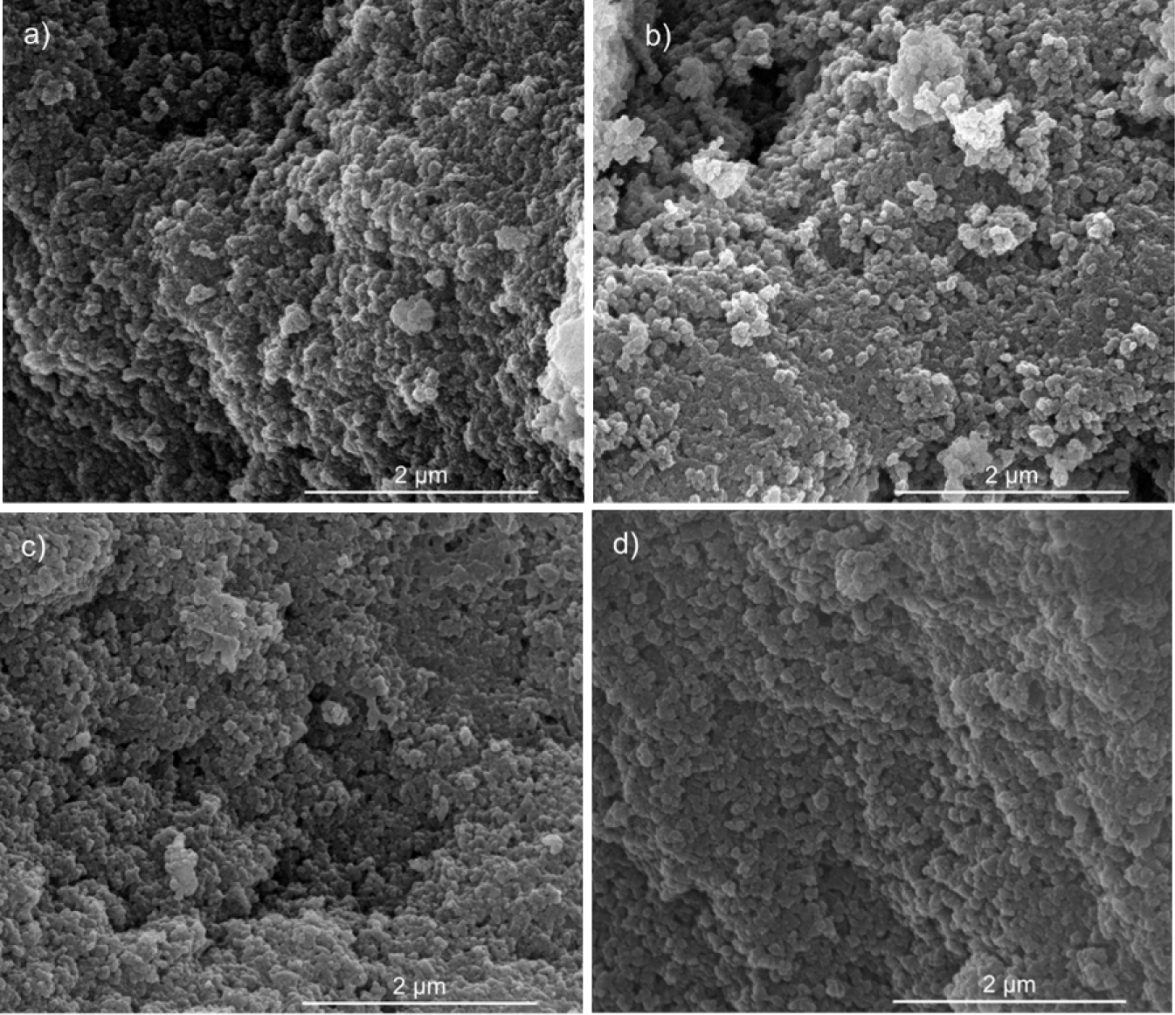
SEM images of fillers (mag 60,000×): (a) SSMMP, (b) SSMMP-NH_2_, (c) SSMMP@PIM-1, and (d) SSMMP-NH_2_@PIM-1.

Visually, SSMMP and SSMMP-NH_2_ appear as well-dispersed white powders (Figure S3a and b). Following the surface deposition of PIM-1 on the filler via the NISD technique, a color change to yellow is evident (Figure S3c and d), attributed to the presence of the benzodioxin unit in the PIM-1 structure, which imparts a fluorescent yellow coloration to the polymer.^68^ These findings indicate that the polymer was deposited on the surface of the SSMMPs.

Transmission electron microscopy (TEM) analysis was also performed to verify the surface deposition of PIM-1, as depicted in Figure 3. The SSMMP-NH_2_ sample (Figure 3a) demonstrated agglomerated spherical particles analogous to those observed in the SEM images (Figure 2). The TEM images revealed overlapping particles, which hindered the clear identification of the structure. Conversely, the SSMMP-NH_2_@PIM-1 sample exhibited the presence of the filler structure, indicating that the PIM-1 surface deposition enhanced particle dispersion. Furthermore, Figure 3b provides additional evidence of the PIM-1 coating on the particle surfaces.

**Figure 3:**
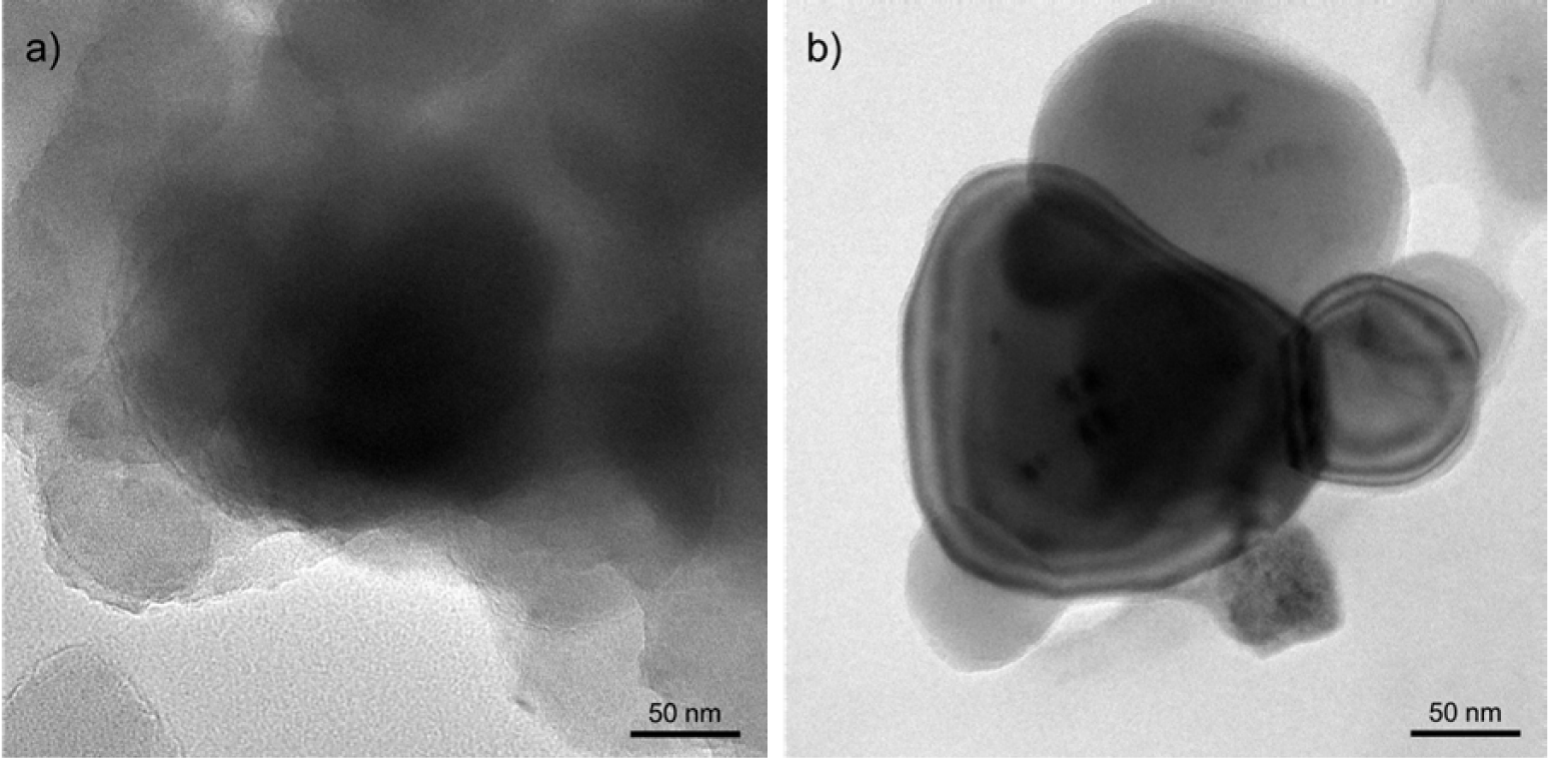
TEM images: (a) SSMMP-NH_2_, and (b) SSMMP-NH_2_@PIM-1 particles.

The FTIR analysis of the fillers is presented in Figure 4. For pure SSMMP, the characteristic bands at 3670 cm^–1^ and 655 cm^–1^ correspond to the stretching vibration of the structural OH groups and to OH libration motions, respectively (Figure 4a), associated with Mg_3_OH species.^69,70^ The wide band between 3650 and 3000 cm^–1^ is related to the −OH of water molecules (Figure 4b), and the band between 1000 and 900 cm^–1^ correlate to the stretching modes of the Si–O bonds (Figure 4c).^69^

**Figure 4:**
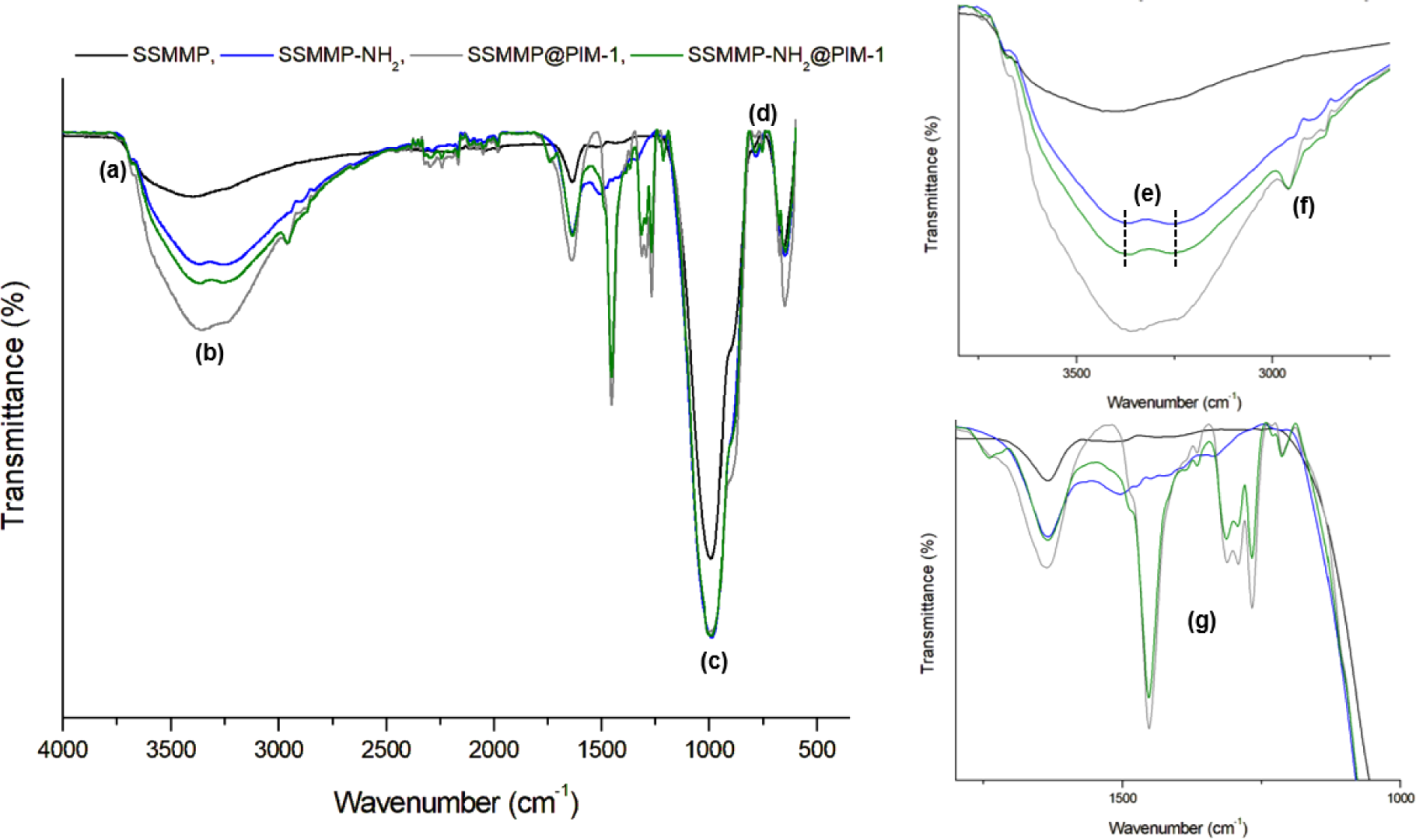
Fourier-transform infrared (FTIR) spectra of SSMMP, SSMMP-NH_2_, SSMMP@PIM-1, and SSMMP-NH_2_@PIM-1.

SSMMP functionalized with −NH_2_ presents new bands at 700 cm^–1^ related to the bending of N–H bonds (Figure 4d),^71^ along with weak bands at 3376 cm^–1^ and 3246 cm^–1^ (asymmetric and symmetric −NH_2_ stretching, respectively) (Figure 4e).^72^ These weak signals are likely due to overlap with the wide band characteristic of hydroxyl groups of water molecules for the pure SSMMP. The increase in intensity at the peak located at 1635 cm^–1^ may be related to −NH_2_.^73^ Furthermore, the C–N stretching vibration, normally observed in 1000–1200 cm^–1^,^71^ is in this case overlapped with the Si–O stretching modes of SSMMP, although the band intensity increased after −NH_2_ functionalization. The presence of −NH_2_ was also verified by the joint presence of nitrogen, Si, Mg, and O in the EDX spectrum (Figure S4).

For core–shell particles with polymer surface deposition, new bands were revealed at the same wavelengths as the PIM-1 bonds (see Figure S2), as can be seen in 2950 cm^–1^ for C–H (Figure 4f), 2239 cm^–1^ for C≡N stretching, and in the region between 1320 and 1250 cm^–1^ for the C–O stretching mode (Figure 4g).

Figure S5 shows the XRD spectra of SSMMP and SSMMP-NH_2_ fillers. An amorphous character of SSMMPs is evidenced by the low intensity and broad diffraction peaks, as previously mentioned in the literature, where there is low stacking and growth in the ab plane.^74,75^ SSMMPs are the precursors of synthetic talc and therefore did not undergo the hydrothermal process for their internal structure to organize into a crystalline structure like talc. After modification of the structure with the incorporation of the −NH_2_ group, the 001 peak became more intense compared to neat SSMMP, minimally increasing the crystallinity along the c direction.^74^ Moreover, after Si was replaced with the amino group, there was no change in the position of the diffraction peaks between SSMMP and SSMMP-NH_2_, indicating that the nanostructure remained unchanged.

TGA of the fillers and PIM-1 is depicted in Figure S6. Pure SSMMP exhibits two characteristic weight loss stages: the first occurs between 25 and 160 °C, assigned to the loss of physisorbed water and reaction solvents. From 160 °C, the TGA appears to show a regular loss. But a more detailed analysis allows us to see two other weight losses: one between 160 °C and about 400 °C, corresponding to the degradation of the Si–OH and Mg–OH groups present on the SSMMP surface,^20^ and the other beyond 400 °C, corresponding to the dehydroxylation of the SSMMP bulk (the SSMMP sample corresponding to the SSMMP-M2 sample of ref. 25). The SSMMP-NH_2_ sample shows a more important weight loss between around 350 and 500 °C, also associated with the degradation of the amino group and the organic structure attached to SSMMP.^76,77^ PIM-1 exhibits weight loss around 100 °C, due to the evaporation of the synthesis solvent, followed by a significant weight reduction at 450 to 700 °C, linked to the decomposition of the polymer backbone and its ether groups.^44,78^ The polymer-coated samples, SSMMP@PIM-1 and SSMMP-NH_2_@PIM-1, display degradation profiles similar to those of the pure fillers. Nonetheless, at 700 °C, it was observed that these coated samples demonstrate lower thermal stability compared to the uncoated counterparts, consistent with the weight loss characteristic of PIM-1, indicating the presence of a certain amount of polymer.

The surface area was measured to evaluate the effect of amino group functionalization and the PIM-1 coating on the filler surface. The results are presented in Table 2. Neat materials presented values comparable to those reported in other studies: 107.4 m^2^/g for SSMMP and 790.9 m^2^/g for PIM-1.^37^ Following amino group functionalization, the surface area of SSMMP-NH_2_ decreased, a phenomenon attributed to the pore-blocking effect induced by the functional groups.^71,73^ In contrast, fillers coated with PIM-1 exhibited an increase in the surface area, which can be ascribed to the substantial free volume of the polymer. Consequently, the shell layer of the filler offers additional interaction sites with CO_2_, thereby establishing a selective pathway for gas transport.

**Table 2:** BET Surface Area of the Filler Materials

Sample	*S*_BET_ (m^2^/g)	Ref
SSMMP	107.4 ± 4.3	This work
SSMMP-NH_2_	87.4 ± 5.2	This work
SSMMP@PIM-1	145.7 ± 9.5	This work
SSMMP-NH_2_@PIM-1	147.3 ± 6.3	This work
PIM-1	790.9 ± 21.2	This work
SSMMP-20%-[bmim][Tf_2_N]	28.2	^21^
SSMMP-5%-Im(nBu)-NTf_2_	151	^25^
SSMMP-Ni 50%	285	^26^
SSMMP-Ni 50%-IMI Br	211	^26^

### 3.3 Membrane Characterization

#### 3.3.1 Dense MMMs

SEM analysis was employed to examine the filler–polymer interaction in MMMs. The surface images are presented in Figure 5, and cross-sectional images in Figure 6. The neat Pebax-2533 membrane was found to be free of defects on its surface and cross-section, as verified in Figures 5a and 6l. For the surface of the MMMs, the filler materials generally exhibit homogeneous dispersion within the polymer matrix, especially at low concentrations (0.5%). However, in the MMMs containing 10% and 20% filler, a tendency toward irregular and uneven surfaces can be observed, particularly in the sample sets (e–f), (h–j), and (k–l), where the filler material appears to agglomerate and form spherical vesicles within the polymer.

**Figure 5:**
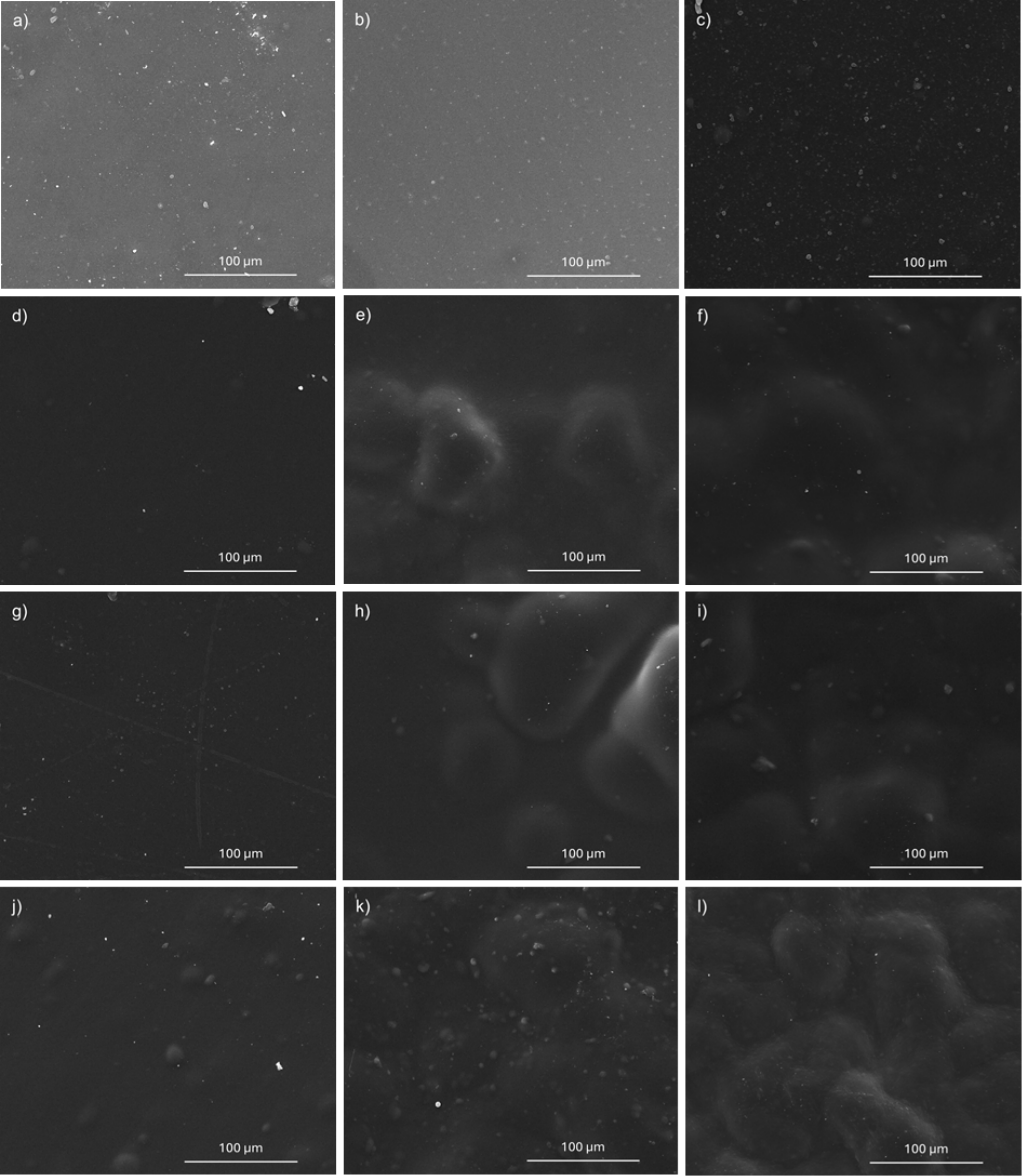
SEM surface images of MMMs: (a) Pebax-2533; (b) and (c) Pebax-SSMMP 0.5 and 20 wt %, respectively; (d), (e), and (f) Pebax-SSMMP-NH_2_ 0.5, 10, and 20 wt %, respectively; (g), (h), and (i) Pebax-SSMMP@PIM-1 0.5, 10, and 20 wt %, respectively; (j), (k), and (l) Pebax-SSMMP-NH_2_@PIM-1 0.5, 10, and 20 wt %, respectively.

**Figure 6:**
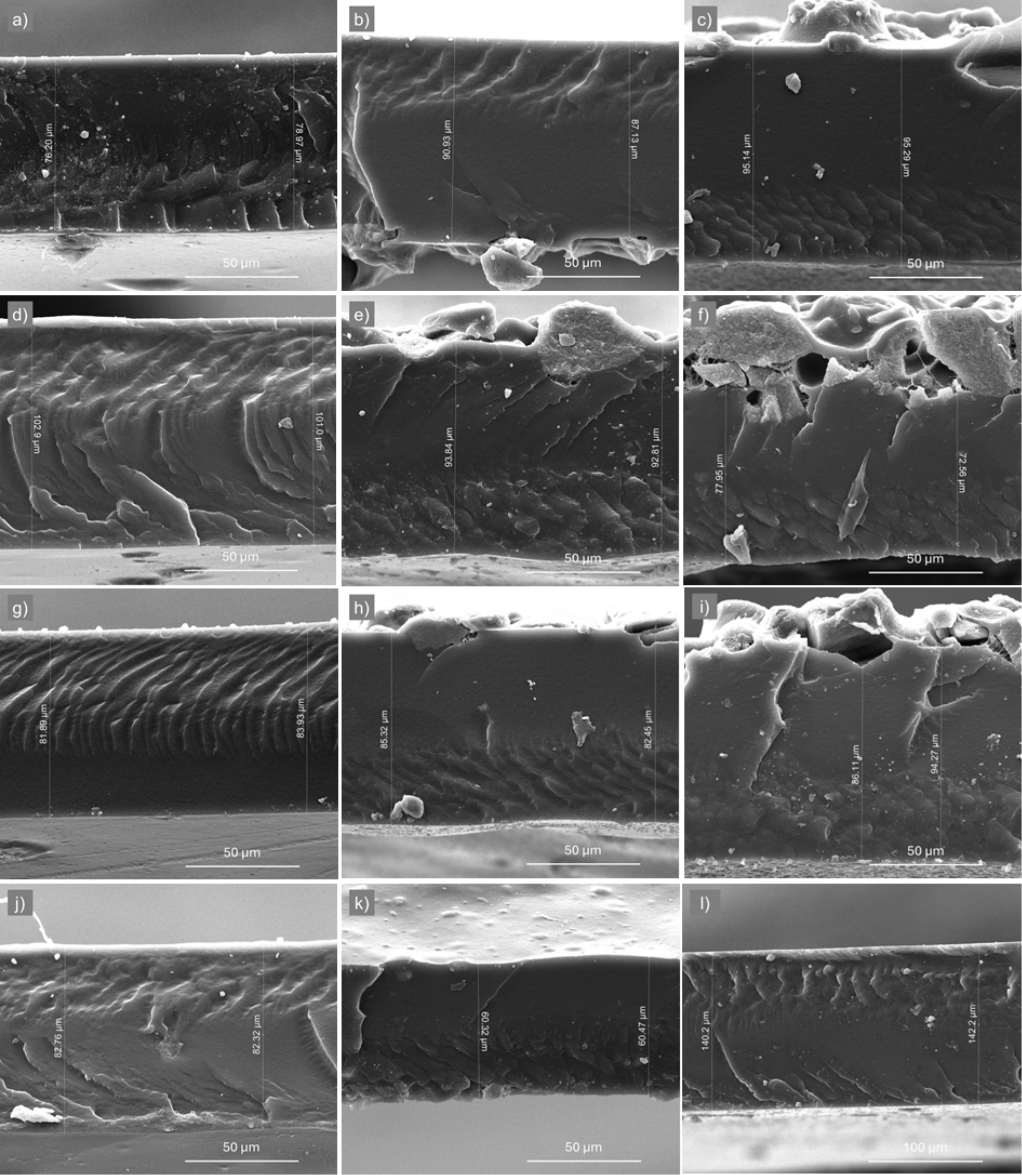
SEM cross-section images of MMMs: (a), (b), and (c) Pebax-SSMMP 0.5, 10, and 20 wt %, respectively; (d), (e), and (f) Pebax-SSMMP-NH_2_ 0.5, 10, and 20 wt %, respectively; (g), (h), and (i) Pebax-SSMMP@PIM-1 0.5, 10, and 20 wt %, respectively; (j) and (k) Pebax-SSMMP-NH_2_@PIM-1 0.5 wt % and 20 wt %, respectively; (l) Pebax-2533.

In the cross-sectional images of the membranes (Figure 6), no pores or nonselective gas pathways were observed. Furthermore, the membranes containing 0.5 and 10 wt % presented a structure similar to that of Pebax-2533, demonstrating good compatibility. The MMMs with 20 wt % of the fillers presented slight changes in the structure, which probably indicate the loading limit of the materials. These structural changes are visible in the upper part of the images in Figure 6f and i, which may be related to the sedimentation of the filler. However, as shown in Figure 6k, the membranes with 20 wt % of the SSMMP-NH_2_@PIM-1 presented good compatibility, probably related to the presence of the amino group and PIM-1 on the filler surface. Thus, the fillers synthesized in this work produce membranes with strong filler/polymer interfacial adhesion, enhancing the gas separation performance. Yet, the cross-sectional images indicate that the thickness of the membranes varied on average between 60 and 100 μm.

The TGA curves of Pebax-2533 and MMMs are illustrated in Figure S7. As shown, the modified membranes demonstrate enhanced thermal stability in comparison with the pure polymer. The initial weight loss of Pebax-2533 occurs at approximately 360 °C,^79^ as a consequence of the rupture of the polymer chains. The MMMs exhibit a higher temperature for weight loss compared to the pure polymer, as evidenced by the *T*_onset_ values presented in Table 3, which indicates that the incorporation of fillers results in membranes with greater thermal stability than neat Pebax.

**Table 3:** Thermal Properties Obtained by TGA Curves of Pebax-2533 and MMMs

Membrane	*T*_onset_ (°C)	*T*_endset_ (°C)
Pebax-2533	357.2	439.7
Pebax-2533/SSMMP 0.5 wt %	359.2	438.2
Pebax-2533/SSMMP 10 wt %	372.3	440.4
Pebax-2533/SSMMP 20 wt %	367.4	438.2
Pebax-2533/SSMMP@PIM-1 0.5 wt %	364.3	440.2
Pebax-2533/SSMMP@PIM-1 10 wt %	367.1	439.9
Pebax-2533/SSMMP@PIM-1 20 wt %	368.4	439.2
Pebax-2533/SSMMP-NH_2_ 0.5 wt %	361.4	439.1
Pebax-2533/SSMMP-NH_2_ 10 wt %	371.3	441.3
Pebax-2533/SSMMP-NH_2_ 20 wt %	367.1	435.5
Pebax-2533/SSMMP-NH_2_@PIM-1 0.5 wt %	370.4	440.7
Pebax-2533/SSMMP-NH_2_@PIM-1 10 wt %	375.0	440.7
Pebax-2533/SSMMP-NH_2_@PIM-1 20 wt %	377.3	438.7

Changes in crystallinity, glass transition temperature (*T*_g_), and melting temperature (*T*_m_) of Pebax-2533 and MMMs were measured by DSC analysis. Table 4 and Figure S8 present the results. The crystallinity of membranes is calculated using eq 5.^80^

5 $$X_{C}=\frac{\Delta H_{m}}{\Delta H_{m}^{o}}$$

**Table 4:** DSC Characterization and Crystallinity of the MMMs Produced in This Work

Membrane	*T*_g_ (°C)	*T*_m,PTMO_ (°C)	*T*_m,PA*‑*12_ (°C)	*X*_C,PTMO_ (%)	*X*_C,PA‑12_ (%)	*X*_C,Total_ (%)
Pebax-2533	–77.6	13.7	135.3	17.1	4.3	14.6
Pebax-2533/SSMMP 0.5 wt %	–76.3	14.6	134.8	14.5	2.3	12.1
Pebax-2533/SSMMP 10 wt %	–76.4	14.3	137.3	10.9	1.8	9.1
Pebax-2533/SSMMP 20 wt %	–75.4	13.8	138.2	7.9	1.6	6.7
Pebax-2533/SSMMP@PIM-1 0.5 wt %	–75.6	14.5	134.8	13.2	1.9	10.9
Pebax-2533/SSMMP@PIM-1 10 wt %	–73.5	13.7	137.1	10.7	1.4	8.8
Pebax-2533/SSMMP@PIM-1 20 wt %	–73.4	13.2	137.6	9.7	1.4	8.1
Pebax-2533/SSMMP-NH_2_ 0.5 wt %	–75.0	12.5	135.1	15.3	1.8	12.6
Pebax-2533/SSMMP-NH_2_ 10 wt %	–74.3	12.6	137.2	12.5	2.0	10.4
Pebax-2533/SSMMP-NH_2_ 20 wt %	–73.0	13.2	137.4	12.8	2.5	10.7
Pebax-2533/SSMMP-NH_2_@PIM-1 0.5 wt %	–74.2	14.3	135.1	13.7	2.9	11.5
Pebax-2533/SSMMP-NH_2_@PIM-1 10 wt %	–72.7	15.5	135.9	10.6	1.7	8.8
Pebax-2533/SSMMP-NH_2_@PIM-1 20 wt %	–70.8	13.8	138.5	7.8	1.3	6.5

Melting enthalpy (Δ*H*_m_) of the respective phases (PTMO or PA-12) is obtained from the peak area using the TA Universal Analysis software, and $\Delta H_{m}^{o}$ is the melting enthalpy of the pure crystalline phase, 167 J/g for PTMO^81^ and 245 J/g for PA-12.^82^ The MMM’s crystallinity was considered with 80% PTMO crystallinity and 20% PA-12 crystallinity, according to Pebax-2533 composition (eq 6).

6 $$X_{Total}=0.8X_{PTMO}+0.2X_{PA-12}$$

Neat Pebax-2533 exhibits a glass transition temperature (*T*_g_) of −77.6 °C and peaks at 13.7 and 135.3 °C, corresponding to the melting temperatures of PTMO and PA-12, respectively, consistent with the findings reported in the literature.^79,83^ Following the incorporation of fillers, the *T*_g_ of the membranes showed a tendency to increase, attributable to hydrogen bonding between the continuous and dispersed phases, which restricted polymer chain mobility.^21^ The rigidification at the filler/polymer interface, as evidenced by the increase in *T*_g_, can be advantageous for enhancing the selectivity of MMMs for gas separation.^84^

The increase in *T*_g_ was more pronounced in samples coated with PIM-1, possibly indicating improved interaction between the filler and the polymer matrix after surface deposition due to better adhesion.^85^ Similar findings were reported by Zhao et al.,^86^ who applied hollow core–shell PIM-1 nanoparticles (PIM HNPs) to Pebax-2533, observing an increase in *T*_g_, and chain rigidification at the interface.

The degree of crystallinity in the MMMs was reduced relative to pure Pebax-2533, owing to the amorphous nature of the fillers (Figure S5), particularly when additionally coated with the amorphous PIM-1 polymer. The decrease in crystallinity may also be linked to the interaction between the carbonyl groups present in the polyamide (PA) structure and the hydroxyl groups,^87^ which reduces interactions with adjacent polymer chains. This phenomenon has been corroborated in studies such as Nobakht’s work^87^ involving the incorporation of maltitol into Pebax 1657, as well as in our prior research,^21^ where SSMMP with ionic liquid was incorporated into Pebax 1657.

Table 5 presents the mechanical properties of the membranes obtained from the stress–strain curves of the DMA analysis. The results were measured in triplicate, with no rupture observed in the samples. The values for Pebax-2533 were similar to those in other works available in the literature.^46,88^

**Table 5:** Mechanical Properties of MMMs Based on Pebax-2533 and SSMMP, SSMMP-NH_2_, SSMMP@PIM-1, and SSMMP-NH_2_@PIM-1 Fillers

Membrane	Young Modulus (MPa)	Stress (MPa)	Strain (%)
Pebax-2533	16.4 ± 0.6	4.6 ± 0.1	185.8 ± 2.9
Pebax-2533/SSMMP 0.5 wt %	24.2 ± 3.9	5.5 ± 0.5	176.3 ± 12.8
Pebax-2533/SSMMP 10 wt %	22.8 ± 6.3	4.0 ± 0.7	186.0 ± 3.3
Pebax-2533/SSMMP 20 wt %	19.4 ± 1.3	3.8 ± 0.3	173.8 ± 7.8
Pebax-2533/SSMMP@PIM-1 0.5 wt %	26.5 ± 2.2	5.0 ± 0.1	170.7 ± 14.0
Pebax-2533/SSMMP@PIM-1 10 wt %	19.0 ± 2.6	5.2 ± 0.7	183.3 ± 2.8
Pebax-2533/SSMMP@PIM-1 20 wt %	16.7 ± 0.1	3.5 ± 0.1	177.0 ± 13.6
Pebax-2533/SSMMP-NH_2_ 0.5 wt %	20.0 ± 1.7	4.8 ± 0.1	184.8 ± 17.4
Pebax-2533/SSMMP-NH_2_ 10 wt %	19.9 ± 1.9	4.3 ± 0.2	178.0 ± 15.4
Pebax-2533/SSMMP-NH_2_ 20 wt %	18.2 ± 0.5	3.1 ± 0.1	151.9 ± 3.4
Pebax-2533/SSMMP-NH_2_@PIM-1 0.5 wt %	18.8 ± 0.9	4.8 ± 0.1	168.0 ± 8.9
Pebax-2533/SSMMP-NH_2_@PIM-1 10 wt %	18.8 ± 1.7	6.4 ± 0.6	176.1 ± 7.4
Pebax-2533/SSMMP-NH_2_@PIM-1 20 wt %	18.4 ± 1.1	4.1 ± 0.2	150.2 ± 9.6

The Young’s modulus exhibited a tendency to increase for all MMMs compared to neat Pebax-2533. It also exhibited a consistent tendency to decrease as the filler concentration increased, likely due to filler agglomeration in the polymer (Figure 5). Although a decreasing trend was observed with increasing filler concentration, the stress–strain modulus of the NH_2_-functionalized membranes remained higher than that of pure Pebax-2533 at all concentrations. This behavior may be attributed to the formation of hydrogen bonds between the NH_2_ groups and the polar groups of the polymer.^89,90^

Additionally, the elongation of the MMMs was lower than that of the pure polymer, indicating that the fillers caused the polymer to stiffen by reducing chain mobility.^88^ This decreased mobility also contributed to higher tensile strength in the MMMs, especially at concentrations of 0.5 and 10 wt %. The samples coated with PIM-1 generally exhibited membranes with higher tensile strength, probably because they better prevented interfacial void formation between the filler and the polymer.

Overall, the mechanical properties improved with the addition of various fillers, likely due to hydrogen bonding between the polar groups of SSMMP (−OH and/or −NH_2_) and the polymer matrix’s polar groups,^89,90^ as well as enhanced filler/polymer interfacial adhesion,^91^ which was further improved by surface deposition of PIM-1.

#### 3.3.2 TFC-MMM

SEM images of the fabricated TFC-MMM (Figure 7) were captured to confirm the successful fabrication of the membrane and to assess the thicknesses of the selective and protective layers. Images of the pure PSF porous support are provided in Figure S9, illustrating a membrane characterized by high porosity on the surface and cross-sectional regions. The CO_2_ permeance of the bare porous support is presented in Note S1. Furthermore, the substrate porosity was estimated by SEM images, which resulted in a porosity of 29.6%, similar to that obtained in the work of Jiang et al.^49^ with the same experimental procedure for porous PSF, which obtained a surface porosity of 26.4%.

**Figure 7:**
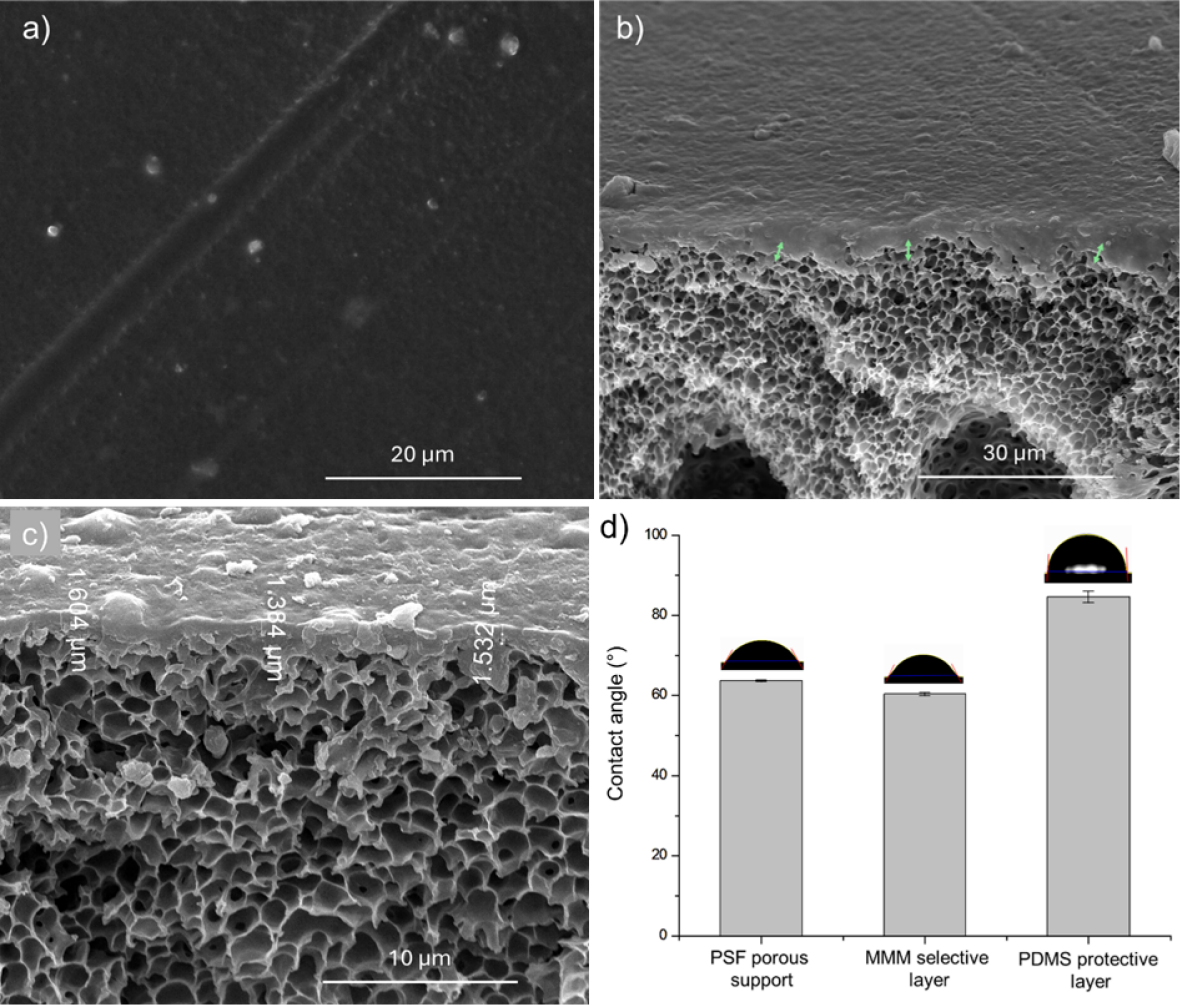
SEM images of surface (a), cross-sectional (b, c) views of TFC based on PSF/Pebax-2533/SSMMP-NH_2_@PIM-1 20 wt %/PDMS, and water contact angle (d) of TFC.

Figure 7a depicts a surface that is dense and free of pores. However, due to the elevated filler loading, some agglomerates may be observed on the surface. This phenomenon occurs because the TFC was prepared using the Pebax-2533/SSMMP-NH_2_@PIM-1 20 wt % sample. Figure 7b presents the cross-sectional view, revealing a thin layer on top of the PSF support. Measuring the TFC selective and protective layers’ thickness separately by cryogenic fracturing was not feasible, since the boundary between the layers was not evident in the cross-sectional images. However, Figure 7c illustrates the maximum thickness of the layers, recorded as 1.5 ± 0.1 μm. Thus, to measure the thickness of the layers separately, the resistance-in-series model was used, as presented in Note S1.

The water contact angle was measured to illustrate the formation of layers in the TFC, as shown in Figure 7d. Pure polysulfone support exhibited a contact angle of 63.6 ± 0.2°, consistent with other studies.^92,93^ Once the Pebax-2533/SSMMP-NH2@PIM-1 layer was introduced, the angle slightly decreased to 60.3 ± 0.4°, reflecting the hydrophilicity of the Pebax polymer matrix.^47,94^ The reduction was modest because PIM-1, which is presented on the surface of the filler, is known as a hydrophobic material.^95,96^ After the protective layer of PDMS was applied, the contact angle increased to 84.6 ± 1.4°, attributable to its hydrophobic properties.

### 3.4 Gas Separation Properties

#### 3.4.1 Dense MMMs

The gas separation properties of the produced MMMs for CO_2_, CH_4_, and N_2_ are presented in Table 6. The analysis was conducted at 4 bar and 25 °C. The neat Pebax-2533 membrane showed a CO_2_ permeability of 167.1 Barrer and ideal selectivities of 13.3 for CO_2_/CH_4_ and 21.4 for CO_2_/N_2_, consistent with other values reported in the literature.^97–99^

**Table 6:** Pebax-2533-Based MMMs Gas Separation Properties at 4 bar and 25 °C

	Permeability (Barrer)			Ideal Selectivity	
Membranes	CO_2_	CH_4_	N_2_	CO_2_/CH_4_	CO_2_/N_2_
Pebax-2533	167.1 ± 1.2	12.5 ± 1.9	7.8 ± 0.5	13.3	21.4
Pebax-2533/SSMMP 0.5 wt %	172.5 ± 1.7	12.9 ± 0.3	7.8 ± 0.3	13.4	22.1
Pebax-2533/SSMMP 10 wt %	221.0 ± 3.2	15.4 ± 0.6	9.9 ± 1.2	14.3	22.3
Pebax-2533/SSMMP 20 wt %	236.6 ± 9.8	15.6 ± 0.4	13.7 ± 0.2	15.1	17.2
Pebax-2533/SSMMP@PIM-1 0.5 wt %	179.2 ± 3.5	13.8 ± 0.6	7.9 ± 0.2	12.9	22.7
Pebax-2533/SSMMP@PIM-1 10 wt %	229.6 ± 1.9	16.7 ± 0.4	11.7 ± 0.3	13.7	19.7
Pebax-2533/SSMMP@PIM-1 20 wt %	294.8 ± 4.0	19.8 ± 0.1	14.4 ± 0.6	14.8	20.4
Pebax-2533/SSMMP-NH_2_ 0.5 wt %	176.7 ± 1.2	12.8 ± 0.2	7.6 ± 0.7	13.7	23.1
Pebax-2533/SSMMP-NH_2_ 10 wt %	271.3 ± 9.1	16.9 ± 0.5	7.8 ± 0.2	16.0	34.8
Pebax-2533/SSMMP-NH_2_ 20 wt %	320.1 ± 6.5	16.6 ± 1.5	8.9 ± 0.0	19.2	36.0
Pebax 2533/SSMMP-NH_2_@PIM-1 0.5 wt %	185.8 ± 2.4	13.5 ± 0.8	8.1 ± 0.2	13.8	22.8
Pebax-2533/SSMMP-NH_2_@PIM-1 10 wt %	292.8 ± 2.5	18.4 ± 1.5	10.4 ± 0.4	15.9	28.1
Pebax-2533/SSMMP-NH_2_@PIM-1 20 wt %	431.1 ± 9.0	26.9 ± 3.7	13.1 ± 0.2	16.1	32.9

In all tested samples, a gradual increase in the CO_2_ permeability was observed with increasing filler concentration. The effect of the filler type on the CO_2_ permeability was also observed: at the same concentration, the fillers SSMMP, SSMMP@PIM-1, SSMMP-NH_2_, and SSMMP-NH_2_@PIM-1, in this order, produced membranes with higher permeabilities. The selectivity of the MMMs for CO_2_/CH_4_, on average, was similar to that of Pebax-2533. In any case, there was an increase in the selectivity proportional to the filler concentration. The same occurred when evaluating the selectivity for CO_2_/N_2_, with more significant increases using the fillers SSMMP-NH_2_ and SSMMP-NH_2_@PIM-1. Minor reductions in selectivity were observed for MMMs with SSMMP and SSMMP@PIM-1 fillers, evident in the CO_2_/N_2_ selectivity of membranes Pebax-2533/SSMMP 20 wt % and Pebax-2533/SSMMP@PIM-1 with 10 and 20 wt %, attributable to filler agglomeration that creates nonselective pathways for gas transport, thereby diminishing membrane selectivity. For Pebax-2533/SSMMP@PIM-1, this hypothesis is corroborated by the progressive increase in N_2_ diffusion with the concentration (Table S1) of the indicated samples.

The samples with the best separation capacities were those with concentrations of 20 wt %, specifically Pebax-2533/SSMMP-NH_2_ 20 wt %, which had a CO_2_ permeability of 320.1 Barrer, with selectivities of 19.2 for CO_2_/CH_4_ and 36.0 for CO_2_/N_2_. Meanwhile, the Pebax-2533/SSMMP-NH_2_@PIM-1 20 wt % sample exhibited a CO_2_ permeability of 431.1 Barrer, along with selectivities of 16.1 for CO_2_/CH_4_ and 32.9 for CO_2_/N_2_. Both membranes demonstrated increases in permeability of 91.6% and 157.9%, respectively, compared with pure Pebax-2533.

The notable enhancement in separation performance of MMMs compared to the pure membrane is likely attributable to the strong interfacial interaction between the fillers and the polymer matrix, as well as the presence of groups with an affinity for CO_2_. The amino group (−NH_2_) is recognized for its capacity to enhance the affinity for CO_2_ in the fillers due to its dipole–quadrupole interaction with carbon dioxide.^77,100^ Furthermore, it can improve the solubility of CO_2_ in the membrane, facilitating its transport.^101^

The incorporation of PIM-1 into the NH_2_-functionalized fillers led to an improved CO_2_ permeability. For both types of fillers, at identical loadings, permeability was consistently higher when PIM-1 was present on the filler surface.

Studies conducted by Kang and collaborators,^34^ in which ZIF-8 and ZIF-8 coated with PIM-1 were incorporated into Pebax-1657, showed that the PIM-1 coating can reduce interfacial defects while simultaneously creating additional and more efficient pathways for gas transport. In our work, the NH_2_-functionalized filler coated with PIM-1 generates a synergistic effect: while PIM-1 primarily contributes to increased CO_2_ diffusion, the NH_2_ groups help maintain the high solubility of CO_2_ (Figure 8). The combination of these effects results in superior performance compared to that achieved with each component individually, and this behavior is most evident in membranes with 20% loading.

**Figure 8:**
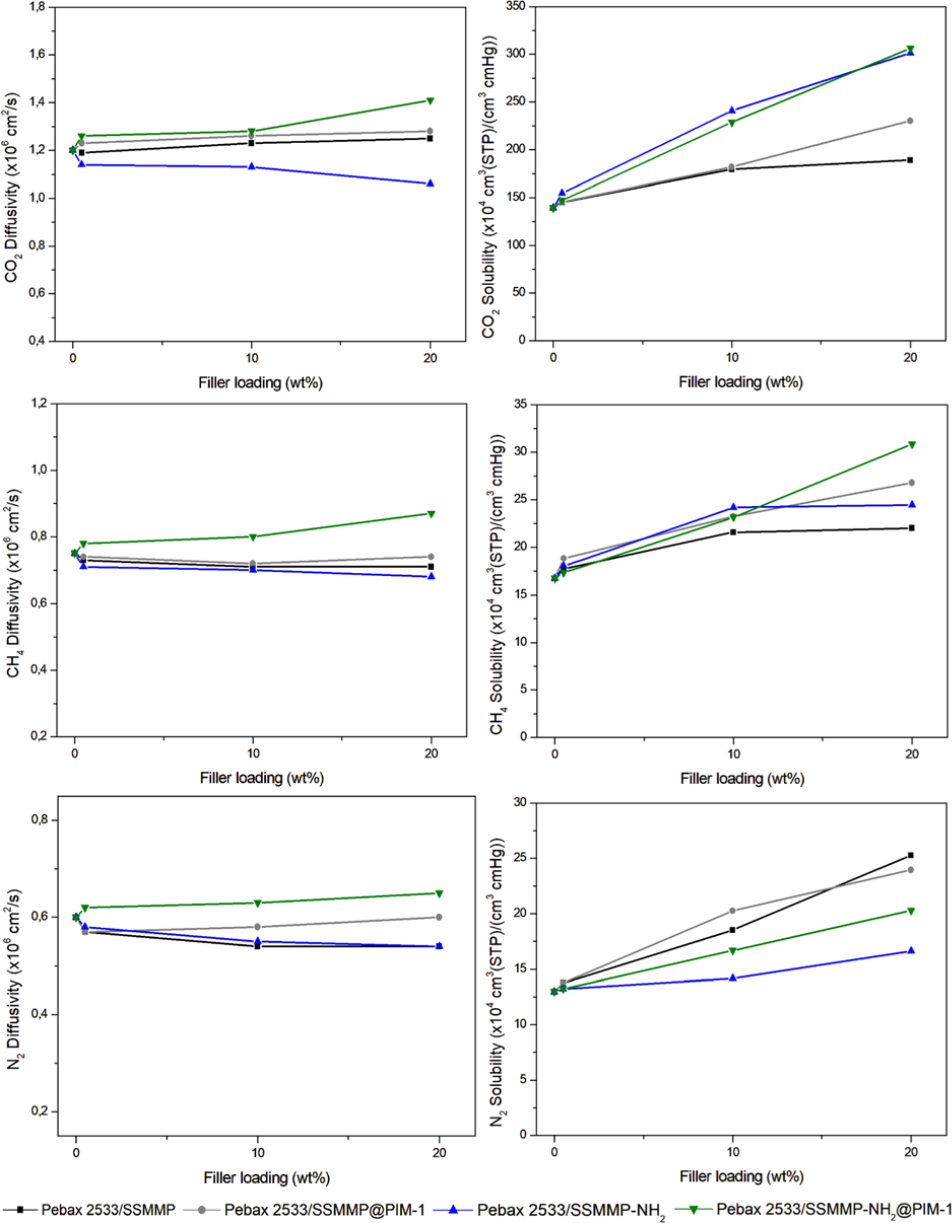
Diffusion and solubility coefficients of CO_2_, CH_4_, and N_2_ for the MMMs incorporating the different filler concentrations (0.5, 10, and 20 wt %): SSMMP (square), SSMMP@PIM-1 (circle), SSMMP-NH_2_ (triangle), and SSMMP-NH_2_@PIM-1 (triangle down).

Membranes containing PIM-1-coated fillers exhibited only a slight decrease in selectivity compared to those with uncoated fillers. This minor decrease is attributed to the increased permeabilities of CH_4_ and N_2_, which could also diffuse through the newly formed permeable pathways.

The solution-diffusion mechanism (eqs 3 and 4) was used to understand the role of fillers and, consequently, the effect of amino groups and PIM-1 in gas separation. The results are presented in Figure 8 and Table S1.

In dense membranes, the gas transport occurs through a solution–diffusion mechanism. For Pebax-2533, this process is predominantly governed by gas solubility, resulting from the strong interaction between CO_2_ molecules and the polar domains of the polymer, particularly the polyether phase.^47^ The flexible PE segments in Pebax-2533 promote efficient CO_2_ transport, and their higher content (80 wt %) in the polymer formulation leads to a greater permeability compared with Pebax-1657, which contains 60 wt % PE.^102^

The incorporation of fillers into Pebax-2533-based membranes in this study positively impacted the solubility and diffusivity coefficients of gas molecules in the mixed matrix membranes, achieving high CO_2_ permeability and ideal selectivity for CO_2_/CH_4_ and CO_2_/N_2_. This increase is associated with the increase in the CO_2_ solubility coefficient, owing to the enhanced CO_2_ adsorption capacity of MMMs attributed to the −OH groups characteristic of the SSMMP structure and primarily to the −NH_2_ group present in the structure of the functionalized fillers.^46,103,104^ Both promote polar-quadrupole interactions with CO_2_, as previously discussed. Taking the Pebax-2533/SSMMP-NH_2_ 20 wt % sample as an example, the CO_2_ solubility was 301.1 [cm^3^(STP)/cm^3^ cmHg × 10^4^], representing a 116% increase compared to the CO_2_ solubility of pure Pebax-2533, which was 139.2 [cm^3^(STP)/cm^3^ cmHg × 10^4^].

When considering the solubility selectivity for the gases presented in Table S1, one can observe an increase in most of the membranes with filler addition compared to that of the pure polymer, particularly in the SSMMP-NH_2_ sample. With a 20 wt % loading, the value obtained was 12.32 for CO_2_/CH_4_ (an increase of 47.9%) and 18.11 for CO_2_/N_2_ (an increase of 68.8%), justifying the higher selectivities found with this material.

The diffusion coefficient of MMMs indicated that after incorporation of the fillers, there was a slight increase in CO_2_ diffusion and a small reduction in the diffusion coefficients of CH_4_ and N_2_ in most of the tested samples. These changes were not significant enough to strongly influence gas separation compared to the alterations in the solubility coefficient, but they may suggest a molecular sieving effect of the fillers.^34,105^ Conversely, MMMs with fillers coated with PIM-1, particularly SSMMP-NH_2_@PIM-1, exhibited increases in the CO_2_, CH_4_, and N_2_ diffusions, likely due to the high gas diffusivity of PIM-1 stemming from the large free volume of its polymer chain.^106^ This characteristic may explain the higher permeabilities and slightly lower selectivities compared to fillers without a polymer coating.

##### 3.4.1.1 Effect of Feed Pressure

The gas feed pressure influence on MMMs was assessed to verify the membrane characteristics as the separation driving force intensifies. With increasing pressure, the membrane’s free volume reduces due to polymer chain compaction,^46^ adversely impacting the diffusion coefficient. In contrast, as CO_2_ is a condensable gas, its solubility enhances with pressure.^98^ Figure 9 illustrates the results for permeability and selectivity, recorded at pressures of 1, 4, 7, and 10 bar at 25 °C for both pure Pebax-2533 and Pebax-2533/SSMMP-NH_2_@PIM-1 20 wt %, chosen by highlighting the optimal separation outcomes.

**Figure 9:**
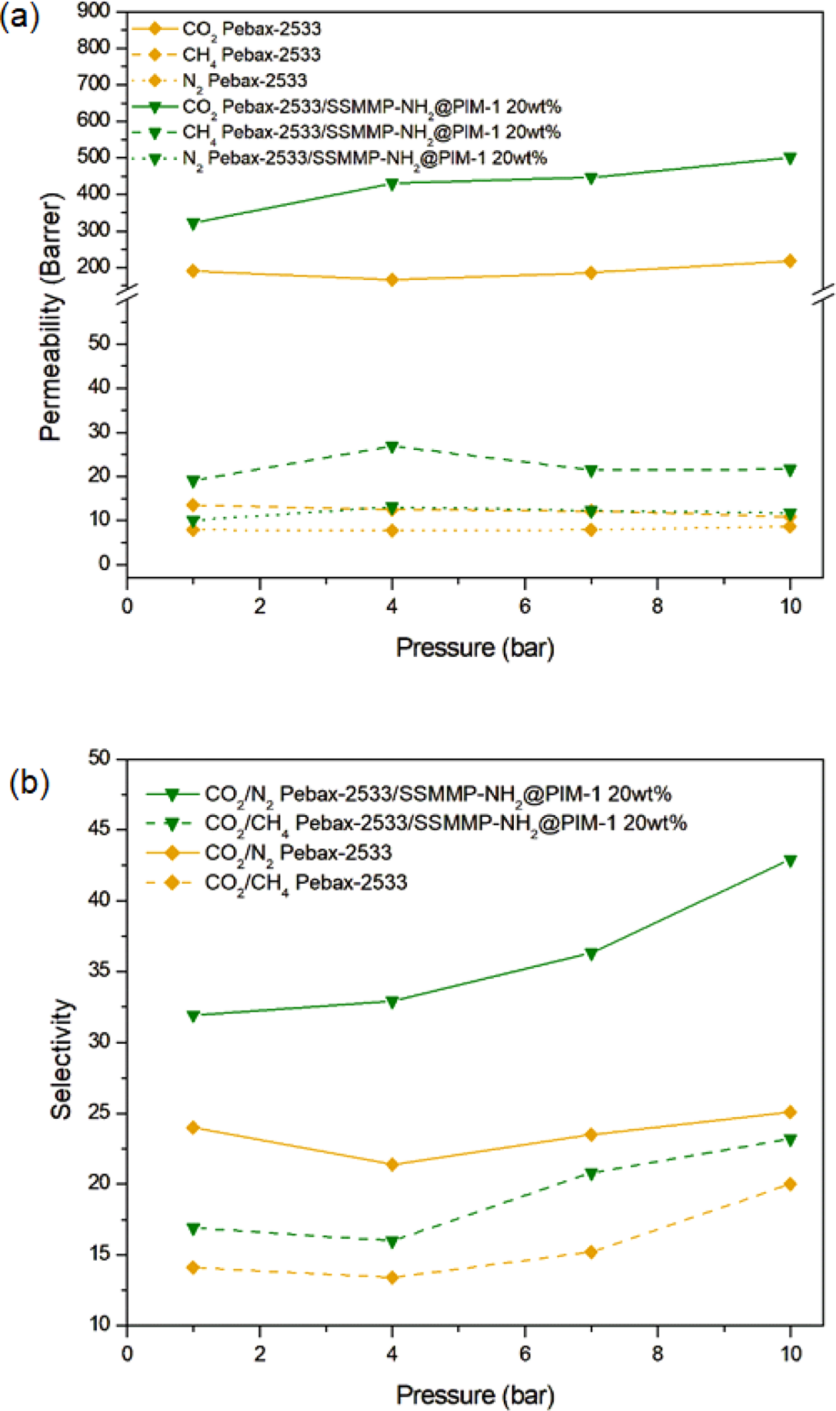
Gas pressure effect on the permeability (a) and CO_2_/CH_4_ and CO_2_/N_2_ selectivity (b).

It was found that the CO_2_ permeability of pure Pebax-2533 experienced a slight reduction from 1 to 4 bar (190.7 to 167.1 Barrer), followed by a gradual increase up to 10 bar (167.1 to 218.3 Barrer), results that are similar to those presented in the literature.^98,99,107^ At higher pressures, the solubility of CO_2_ in the polar ether groups of Pebax leads to increased permeabilities.^99^ For Pebax-2533/SSMMP-NH_2_@PIM-1 20 wt %, a gradual increase in CO_2_ permeability (322.4 to 501.7 Barrer) was observed, this time attributed not only to the high affinity of CO_2_ in the polymer matrix but also to the enhanced CO_2_ solubility in the −NH_2_ functionalized filler. Furthermore, the MMM demonstrated improved results compared to the pure membrane at all of the studied pressures.

The membrane selectivity also increased slightly with the pressure. This occurred because CH_4_ and N_2_ are less condensable gases. Thus, the solubility of CH_4_ and N_2_ gases decreases with increasing pressure.^108^ For these gases, the permeability does not increase significantly with pressure.

##### 3.4.1.2 Robeson Upper Bound and Literature Comparison

The gas separation results were evaluated at the Robeson upper bound^7,8^ and the 2019 trade-off curve presented by Comesaña-Gándara^109^ and compared with values communicated in the literature, as shown in Figure 10 and Table S2, respectively.

**Figure 10:**
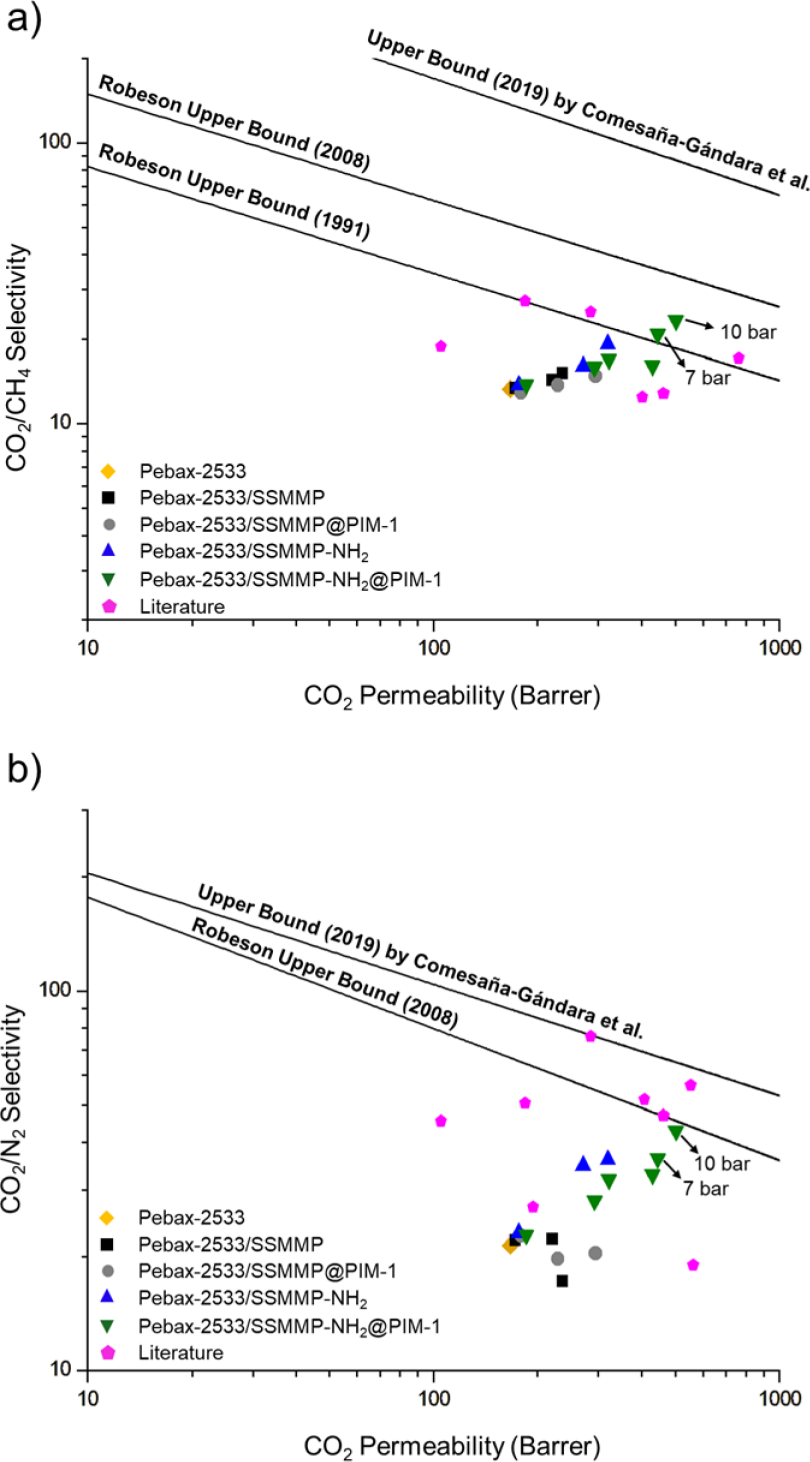
Comparison of CO_2_/CH_4_ (a) and CO_2_/N_2_ (b) separation performance of MMMs produced in this work. Pebax-2533/SSMMP-NH_2_@PIM-1 presents samples with 0.5, 10, and 20 wt % concentrations, measured at 4 bar (Table 6), as well as samples with 20 wt % concentration measured at 1, 7, and 10 bar (Figure 9). Literature materials are described in refs. 34, 42, 46, 97, 110–115 and presented in Table S2.

For CO_2_/N_2_, the membranes performed below the Robeson curve. However, results measured at 7 and 10 bar for the sample with 20 wt % of SSMMP-NH_2_@PIM-1 indicate that the separation capacity closely approached the trade-off. Although the membrane permeability increased significantly, the corresponding increase in selectivity was insufficient, given that Pebax-2533 inherently exhibits low gas selectivity. Regarding CO_2_/CH_4_, the mixed matrix membranes (MMMs) with 20 wt % of SSMMP-NH_2_@PIM-1 tested at 7 and 10 bar surpassed the 1991 curve, while the MMMs with 20 wt % SSMMP-NH_2_ and SSMMP-NH_2_@PIM-1 tested at 4 bar were close to this benchmark. Additionally, the graph reveals a consistent pattern among the samples, with both permeability and selectivity exhibiting a continuous increase proportional to the filler concentration, thereby projecting toward the trade-off curves.

Overall, the fillers produced in this work, especially SSMMP-NH_2_ and SSMMP-NH_2_@PIM-1, achieved comparable or superior separation performance when compared to previous studies utilizing Pebax-2533 as the continuous phase (Table S2). The permeability for CO_2_ in membranes examined in this study exceeded most results reported in the literature, along with the ideal CO_2_/CH_4_ selectivity, which was marginally higher compared to that of alternative materials. Conversely, the selectivity for CO_2_/N_2_ was lower than the other values. Therefore, based on the findings and the comparative analysis with the literature, the most effective membrane fabricated was Pebax-2533 with 20 wt % SSMMP-NH_2_@PIM-1, tested at 10 bar, exhibiting a permeability of 501.7 Barrer and selectivities of 23.2 for CO_2_/CH_4_ and 42.9 for CO_2_/N_2_.

##### 3.4.1.3 Physical Aging of Membranes

To assess the impact of PIM-1 physical aging on the surface of SSMMPs, the MMM containing 20 wt % SSMMP-NH_2_@PIM-1 was subjected to permeability testing after 200 days. For comparative purposes, the MMM with 20 wt % SSMMP-NH_2_ was also analyzed. The results, depicted in Figure 11, demonstrate a reduction in the level of CO_2_ permeability in the MMM with the PIM-1-coated filler and a slight decrease, although statistically insignificant, in the MMM with the uncoated filler.

**Figure 11:**
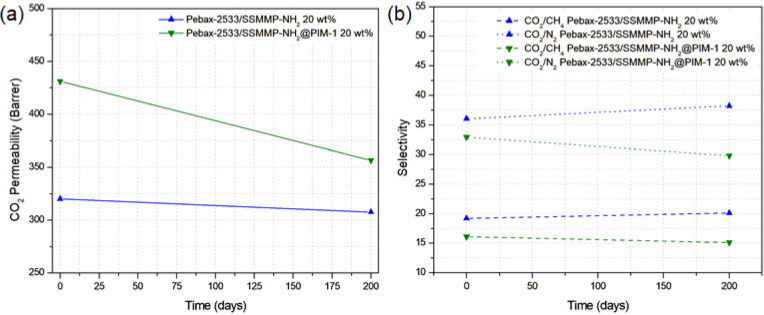
Physical aging for MMMs incorporated with SSMMP-NH_2_@PIM-1 and SSMMP-NH_2_ after 200 days: (a) CO_2_ permeability, and (b) CO_2_/CH_4_ and CO_2_/N_2_ selectivity.

Research within the literature indicates the long-term stability of membranes based on Pebax-2533. The investigation by Qin et al.^107^ assessed Pebax-2533 MMMs integrated with SUM-1 and SUM-9, revealing that, after a period of 30 days, both CO_2_ permeability and CO_2_/N_2_ selectivity remained unaffected under humid conditions. Similarly, Lee et al.^116^ fabricated Pebax-2533 membranes incorporating KTFSI, confirming the stability of CO_2_ permeance and CO_2_/N_2_ selectivity over 860 h. Wei et al.^117^ addressed a strategy for preparing Pebax-2533 membranes via nonsolvent-induced microstructure rearrangement (MSR), demonstrating long-term stability for CO_2_/N_2_ separation over a duration of 7 days. Thus, based on information reported in the literature and the aging results obtained in this study, in which MMM containing pure SSMMP-NH_2_ did not show a loss of separation capacity over time, the reduction in permeability after 200 days can be attributed to the presence of PIM-1 polymer on the filler surface.

PIM-1 undergoes structural densification over time,^118^ thereby decreasing gas permeability through the partial collapse of its microporosity.^119^ The physical aging of the PIM-1 coating on the filler restricts the permeable separation capacity at the interface between the polymer and the filler, as PIM-1 enhances gas diffusion and consequently increases the overall membrane permeability. Thus, after the densification of the PIM-1 structure, MMM acquires a plugged sieve-like characteristic—known for decreasing membrane permeability^120^—due to the reduction of the polymer fractional free volume. Conversely, the CO_2_/CH_4_ and CO_2_/N_2_ selectivities of the MMMs remained virtually unchanged following the aging process. Although the overall physical aging negatively affected the membrane characteristics with PIM-1-coated filler, the permeability loss after an extended period of 200 days continued to allow for substantial gas separation performance, exhibiting a smaller decrease in permeability compared with studies utilizing PIM-1 as the MMM polymer. Consequently, this research presents a novel alternative for the application of PIMs.

#### 3.4.2 TFC-MMM

The TFC membrane development is essential from an industrial perspective, as the selective thin film enables high separation flux and is supported by a porous substrate that offers the requisite mechanical stability.^15,121^ Consequently, TFCs were fabricated in this study to assess the separation performance of the synthesized materials under conditions more representative of typical gas separation applications. To this end, a thin film of the most suitable dense sample (Pebax-2533/SSMMP-NH_2_@PIM-1 20 wt %) was prepared on a porous polysulfone support, and its permeance for CO_2_, CH_4_, and N_2_ was measured at 4 bar and 25 °C. The results are summarized in Table 7 along with comparable data from the literature and in Figure 12, which represents the target area proposed by Merkel et al.,^122^ where TFCs must achieve CO_2_ permeances greater than 1000 GPU, with CO_2_/N_2_ selectivities superior to 20.

**Table 7:** Pure Gas Properties of Pebax-2533/SSMMP-NH_2_@PIM-1 20 wt % TFC Membrane at 4 bar and 25 °C, and Literature TFCs Results

	Permeance (GPU)			Ideal Selectivity		
Membranes	CO_2_	CH_4_	N_2_	CO_2_/CH_4_	CO_2_/N_2_	Ref
PSF/Pebax2533/SSMMP-NH_2_@PIM-1	621 ± 55	77 ± 12	32 ± 9	8	19	This work
PSF/Pebax2533/SSMMP-NH_2_@PIM-1/PDMS	575 ± 41	48 ± 4	17 ± 5	12	33	This work
PAN/Pebax-2533	385				24	^123^
Pebax 2533/PEI	48.2		1.7		29	^124^
PAP/IL/ZIF-20%	1208	66	139	9	19	^50^
PAN/Pebax2533-HMA-PEO	1070				22	^125^
Pebax1657/MX-0.05	1986.5	134.2	47.5	14.8	41.8	^126^
Pebax1657/ZIF-8 30 wt %	350			13	31	^127^
PTO-U30	1828	132	56	13.8	32.4	^51^
Pebax 1657/TA-ZIF-8/TCOH 0.184 wt %	1178.56				63.13	^128^
Pebax 1657/TA-ZIF-8/TCOH 0.184 wt %	1097.62			58.66		^128^
PZ-1	291			23	68	^129^
PAN/Pebax-2533/PEGb-PDMS	1030				21	^130^
PIM-1/CuBDC-ns	407.3			15.6		^131^
PSF/PTMSP/Pebax/*br*-NH_2_-*h*-ZIF-8–10 wt %	833				53.1	^132^
Pebax-1657/PDMS–PEO/PAN	2142				36	^133^
Pebax-1657/ZnTCPP/PAN	1710				34	^134^
PSF/PTMSP/r-200%PEBA	2371		52.8		44.9	^135^
Pebax-PPEGMEA/PDMS–PEG/PEI	271				32	^136^

**Figure 12:**
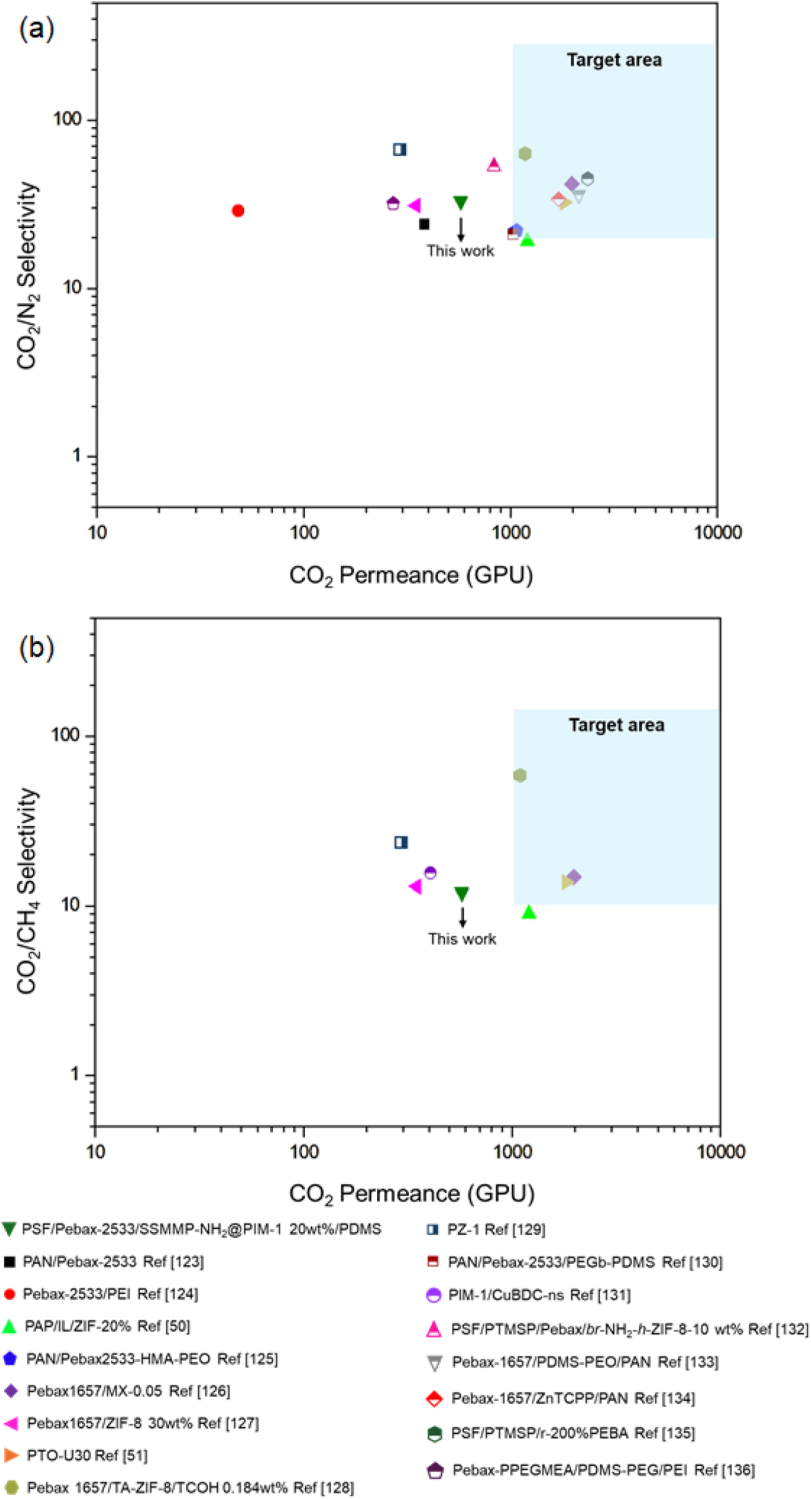
CO_2_/N_2_ (a) and CO_2_/CH_4_ (b) separation performance of the TFC membranes in this work compared to TFC membranes reported in the literature.

The fabricated TFC attained a permeance of 575 GPU for CO_2_, with selectivities of 12 for CO_2_/CH_4_ and 33 for CO_2_/N_2_. Generally, the findings were consistent with those documented in the literature (Figure 12); however, they did not reach the targeted region, as the permeance was lower than that reported for other membrane data. Nevertheless, the CO_2_ permeance outcomes of this study demonstrate enhancements relative to previously reported values, such as those associated with pure Pebax-2533, fabricated on polyacrylonitrile (PAN) support with a PDMS gutter layer, as developed by Tan et al.^123^ Moreover, the findings surpass those reported by Sutrisna et al.,^127^ in the context of preparing hollow fiber membranes of Pebax-1657 with 30 wt % of ZIF-8.

Conversely, the CO_2_/N_2_ selectivity is more favorable compared to those of other reported materials. It is likely that the PDMS protective layer mitigated defects and enhanced selectivity by obstructing nonselective pathways. This is corroborated by the slightly higher CO_2_ permeance of TFC-MMM without the PDMS protective layer, but with considerably lower selectivity, as shown in Table 7. Furthermore, other studies in the literature, as presented in Table 7, do not incorporate a protective layer; rather, they utilize only the gutter layer to standardize the selective layer. To corroborate this hypothesis, a TFC membrane was fabricated by depositing only a thin PDMS layer onto PSF porous support, following the experimental procedure described in Section 2.5.2. The permeance values for CO_2_, CH_4_, and N_2_ are presented in Table S3 and are consistent with other studies in the literature, as presented in refs. 137 and^138^. The low ideal selectivity observed in the PSF–PDMS indicates that the applied protective layer on TFC presented in Table 7 acts mainly by correcting the defects of the selective layer without a significant influence on gas transport.

Another aspect that may have contributed to the good results was the use of SSMMP-NH_2_ nanoparticles coated with PIM-1, since small-sized materials are more suitable for producing TFC-MMM. The findings suggest that the synthesized TFC has potential avenues for enhancement, including the utilization of other porous supports.

Nonetheless, the performance of the TFCs developed in this study is lower than that reported for comparable materials in the literature, as shown in Figure 12, especially in comparison to TFC membranes fabricated with a Pebax-1657 selective layer, given the superior selectivity typically achieved with this material. A plausible explanation for this behavior is the type and quality of the porous support used, which was produced in the laboratory and may therefore exhibit lower uniformity and a higher propensity for defect formation compared to commercial supports. Additionally, several studies in the literature employ PAN supports, which are considered the benchmark for TFC fabrication. Another contributing factor may be the partial penetration of the selective layer into the pores of the PSF support, increasing resistance for gas transport and consequently reducing gas permeance, suggesting the need for a gutter layer to prevent penetration.

## 4 Conclusion

This study incorporated SSMMP fillers modified with amino groups (−NH_2_) and coated with PIM-1 into the Pebax-2533 polymer matrix to create MMMs with improved gas separation performance. The highly polar filler formed hydrogen bonds with the polymer, boosting the membranes’ thermal and mechanical properties through improved filler/polymer interfacial interaction, as verified by the changes in *T*_g_ values, which had increased in all MMMs, particularly for the membranes containing 20 wt % SSMMP-NH_2_ and SSMMP-NH_2_@PIM-1, which exhibited glass transition temperatures of −73.0 °C and −70.8 °C, respectively, compared to −77.6 °C for neat Pebax-2533. The compatibility between the continuous phase and the dispersed phase was further enhanced by the PIM-1 coating, as confirmed by the permeability tests. The most effective sample for gas separation contained 20 wt % SSMMP-NH_2_@PIM-1, achieving CO_2_ permeability of 431.1 Barrer, CO_2_/CH_4_ selectivity of 16.1, and CO_2_/N_2_ selectivity of 32.9 at 4 bar. Tests showed that both permeability and selectivities increased with feed pressure, reaching the Robeson upper bound for CO_2_/N_2_ and surpassing it for CO_2_/CH_4_ at a 10 bar pressure. Additionally, a TFC membrane fabricated using this optimal dense membrane configuration as a selective layer showed a CO_2_ permeance of 575 GPU, selectivity of 12 for CO_2_/CH_4_, and selectivity of 33 for CO_2_/N_2_. The results obtained in this study highlight the potential of SSMMP-based fillers, an emerging class of materials in MMM research, whose structural characteristics enable functional group modifications to enhance the CO_2_ affinity. Here, both the functionalization of −NH_2_ groups and the application of a PIM-1 coating contributed to membranes with an improved performance capability. Another relevant outcome of this work is the successful application of the NISD technique to produce core–shell particles, thereby expanding the scope previously explored by other works.^34^ Despite these advances, further detailed studies are required to enhance the separation performance achieved. For TFC membranes, in particular, future work can explore the incorporation of a gutter layer and the use of PAN porous supports. Additionally, investigating polymer matrices with higher selectivity for gas mixtures represents another promising direction for their continued development. Furthermore, future works with mixed-gas permeation measurements, accounting for the competitive sorption effects, would provide a more realistic evaluation of the separation performance of the developed membranes.

## Supplementary Material



## Notes

Additional experimental details include the priming method (Figure S1), supplementary figures depicting the characterizations of the synthesized materials (Figures S2 to S9), diffusion and solubility coefficients (Table S1), a comparison with literature data (Table S2), and general information regarding TFC membranes (Table S3 and Note S1) (PDF)
