# Supplementary material for: Optimizing SSMMP-Based Fillers with −NH2 Functionalization and PIM‑1 Coating for High-Performance CO2/CH4 and CO2/N2 Separation in Mixed Matrix Membranes and Thin-Film Composite (TFC) Membrane
Source: ACS Omega. 2026 Jan 22;11(4):6477–99. doi: 10.1021/acsomega.5c11506 (PMC12878510; doi:10.1021/acsomega.5c11506)
Supplement: Supplementary file 1 [file ao5c11506_si_001.pdf]

Optimizing SSMMP-based fillers with -NH_2 functionalization and PIM-1 coating for high-performance CO_2/CH_4 and CO_2/N_2 separation in mixed matrix membranes and thin-film composite (TFC) membrane

Henrique Z. Ferrari [a*], Christophe Le Roux [b], Franciele Bernard [a, c*], Guilherme Dias [a], Leonardo dos Santos [c], Pierre Micoud [b], Stéphane Mazières [d], François Martin [b], Sandra Einloft [a, c]

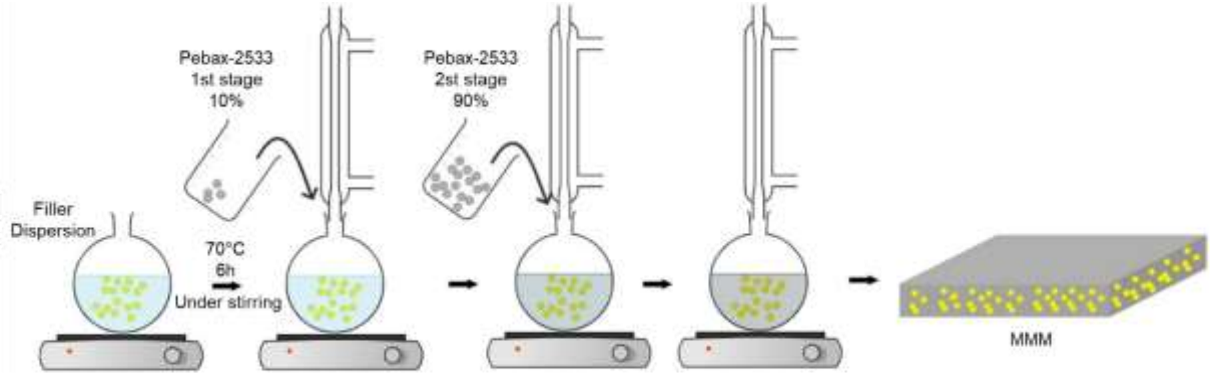

Figure S1. Priming method flowchart.

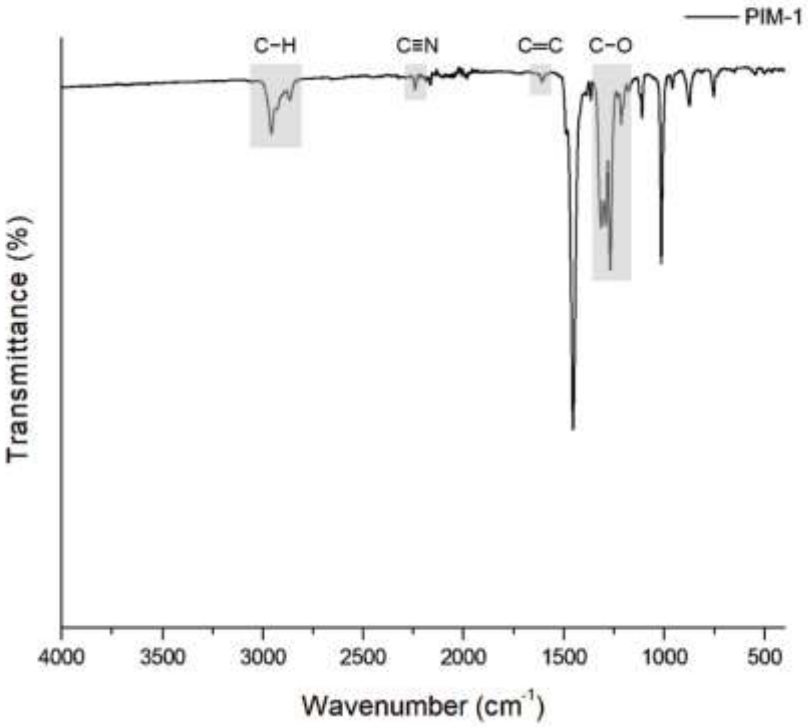

Figure S2. FTIR spectra of PIM-1.

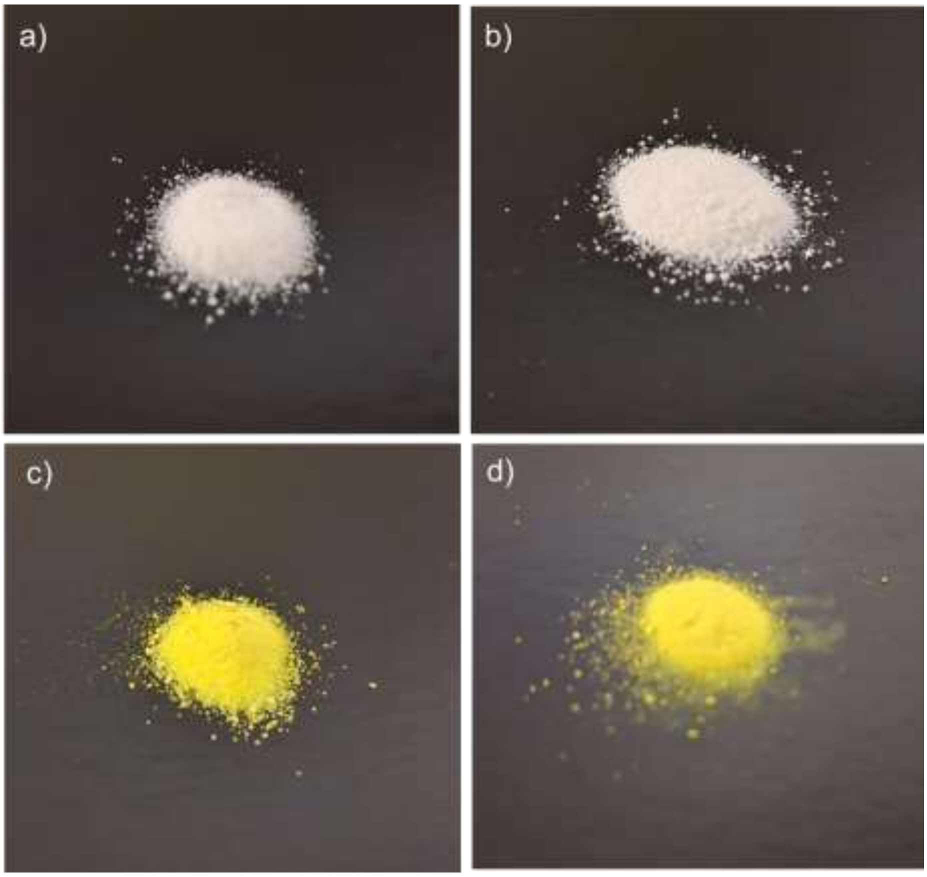

Figure S3. Photos of fillers: a) SSMMP, b) SSMMP-NH_2, c) SSMMP@PIM-1, and d) SSMMP-NH_2@PIM-1.

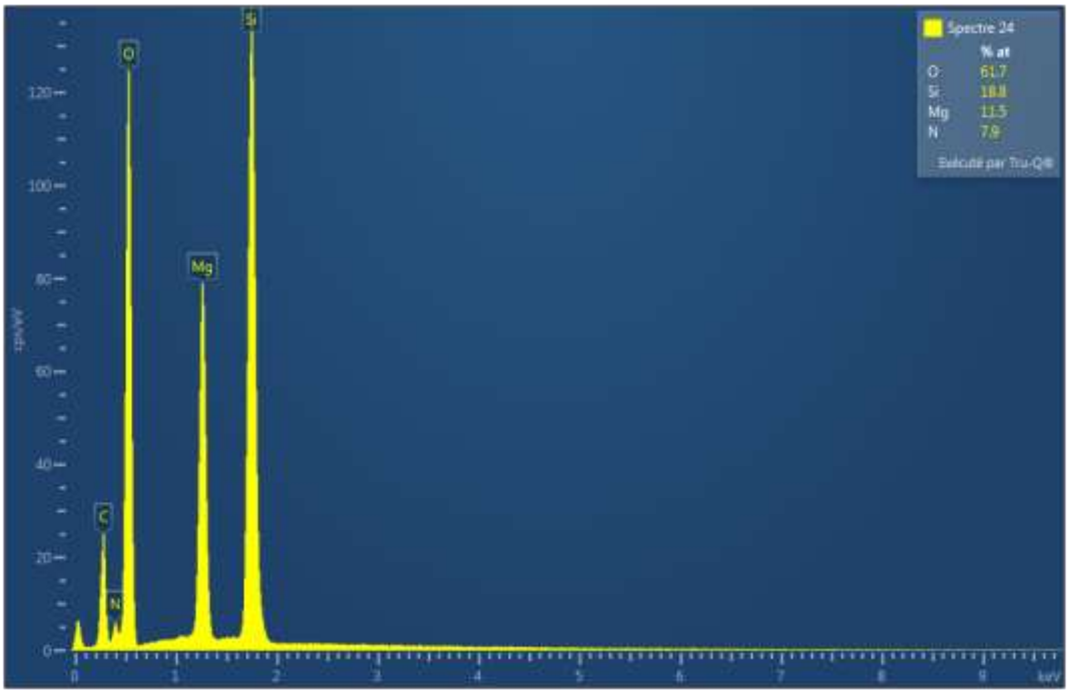

Figure S4. EDX spectrum of SSMMP-NH_2.

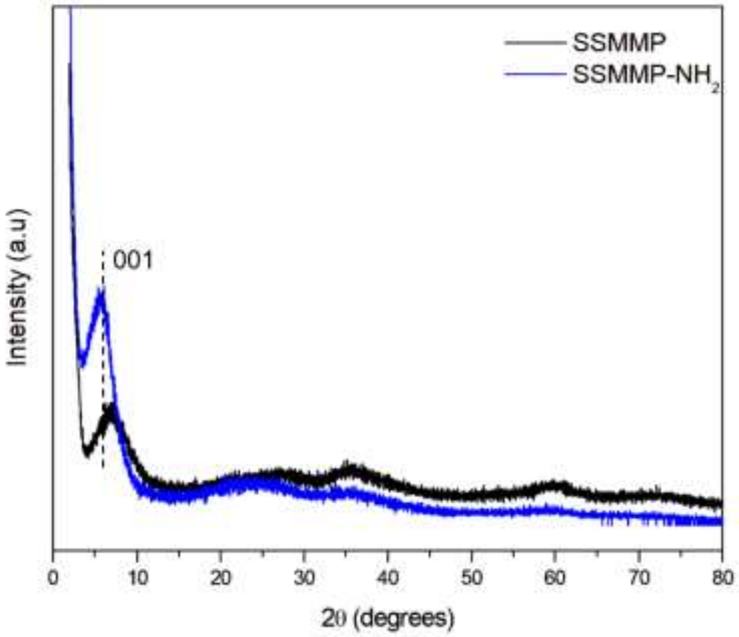

Figure S5. XRD patterns of SSMMP and SSMMP-NH$_2$.

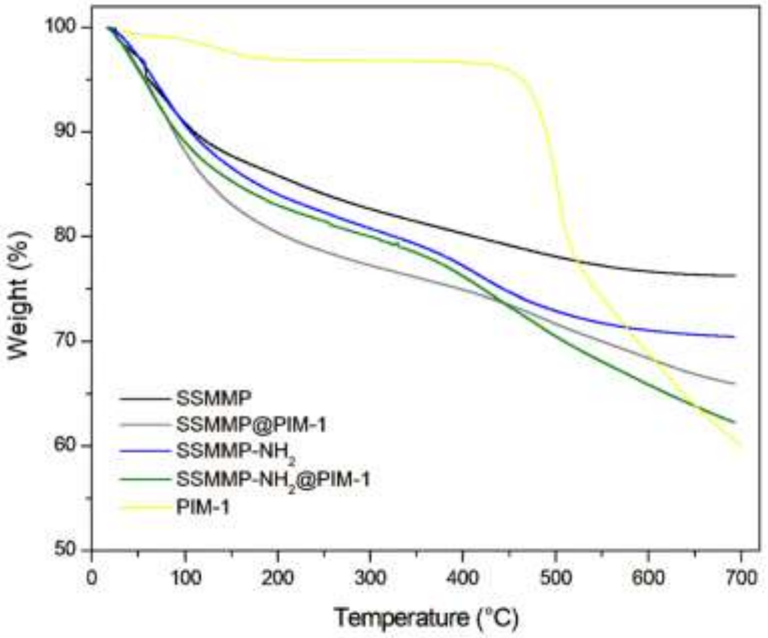

Figure S6. TGA curves of SSMMP, SSMMP-NH$_2$, SSMMP@PIM-1, SSMMP-NH$_2$@PIM-1, and PIM-1 polymer.

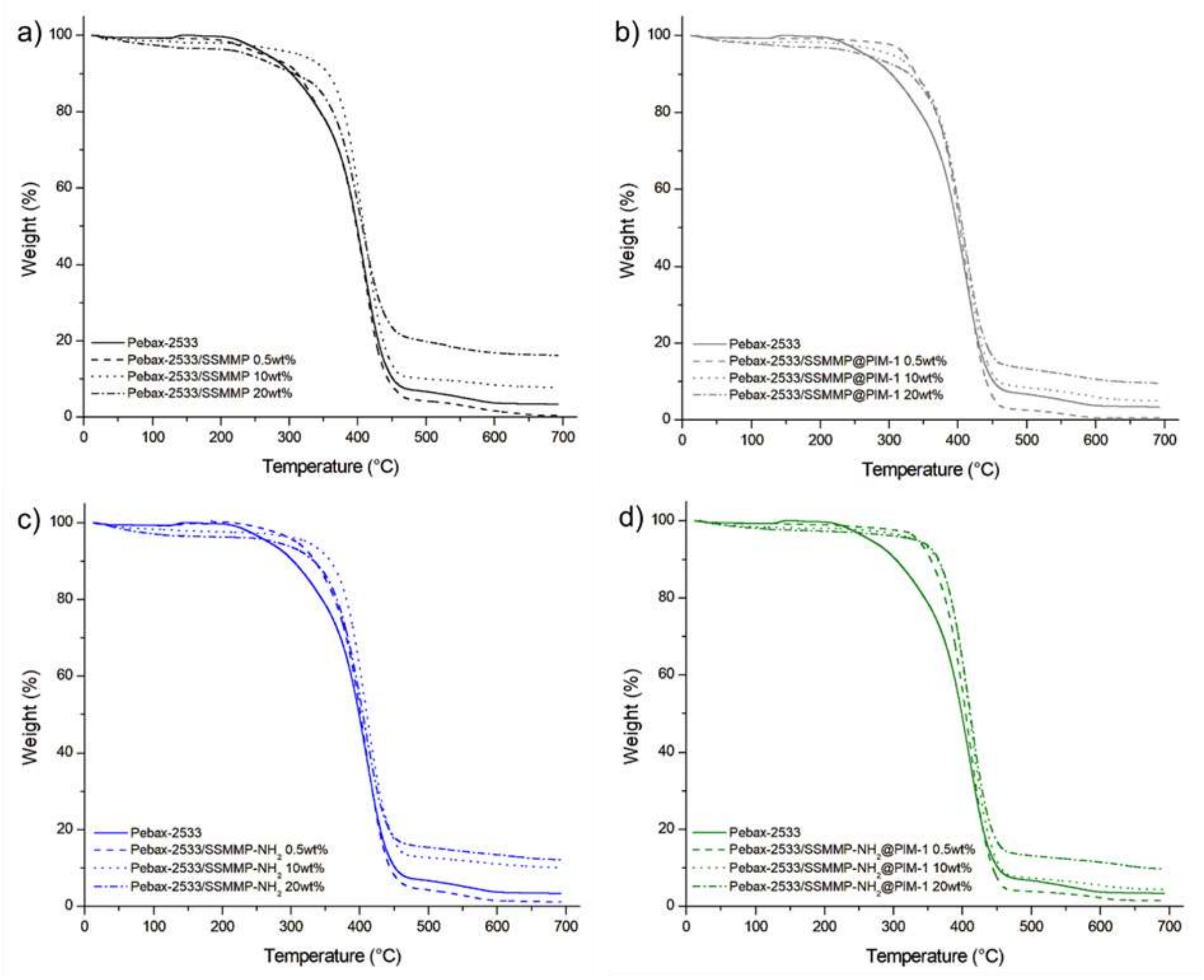

Figure S7. TGA curves of Pebax-2533 based MMMs with fillers: a) SSMMP, b) SSMMP@PIM-1, c) SSMMP-NH_2, and d) SSMMP-NH_2@PIM-1

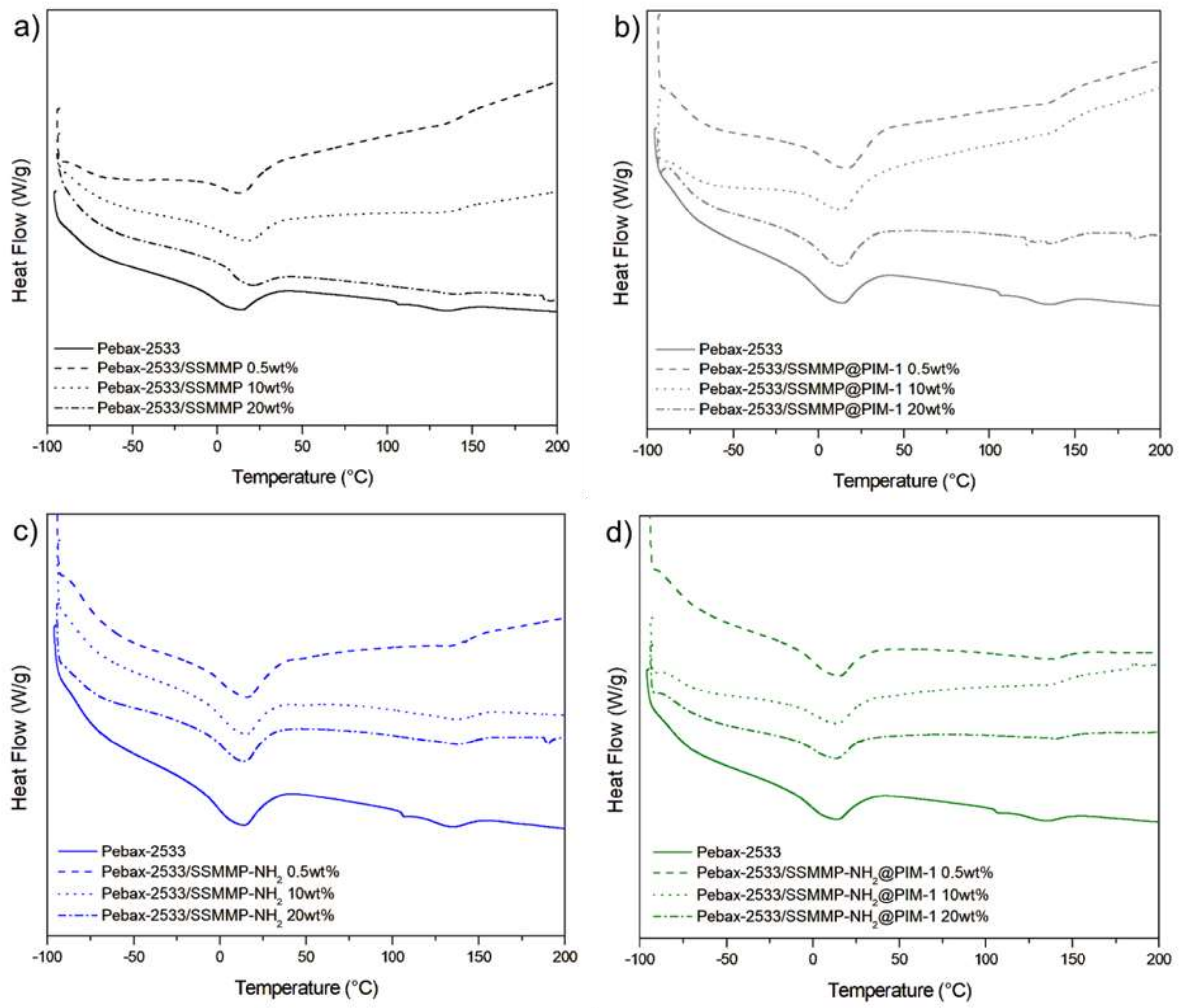

Figure S8. DSC thermograms of Pebax MMMs: a) SSMMP, b) SSMMP@PIM-1, c) SSMMP-NH_2, and d) SSMMP-NH_2@PIM-1

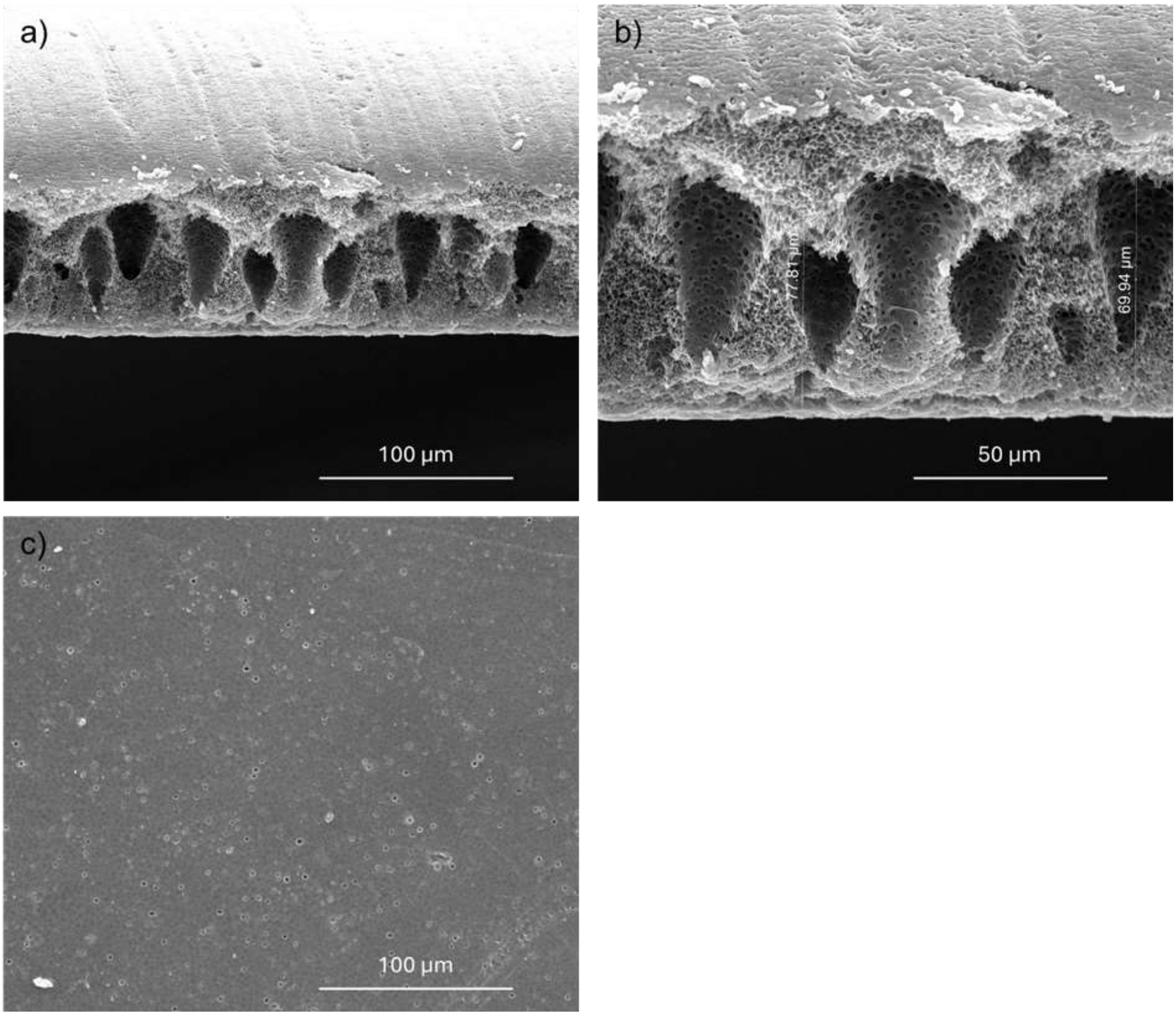

Figure S9. SEM images of pure PSF porous support for TFC membrane

Table S1. Diffusion and solubility coefficients of Pebax 2533 based MMMs (4 bar, 25 °C).

Membrane	D_{CO_2} [a]	D_{CH_4} [a]	D_{N_2} [a]	S_{CO_2} [b]	S_{CH_4} [b]	S_{N_2} [b]	D_{CO_2}/D_{CH_4}	D_{CO_2}/D_{N_2}	S_{CO_2}/S_{CH_4}	S_{CO_2}/S_{N_2}
Pebax-2533 [c]	1.80	1.31	2.05							
Pebax-2533 [d]	≈2.20	≈1.91	≈1.60	≈149.4	≈20.3	≈5.90	≈1.16	≈1.38	≈7.49	≈25.60
Pebax-2533	1.20	0.75	0.60	139.2	16.7	12.9	1.60	1.99	8.33	10.73
Pebax-2533/SSMMP 0.5 wt%	1.19	0.73	0.57	145.1	17.7	13.7	1.63	2.10	8.19	10.56
Pebax-2533/SSMMP 10 wt%	1.23	0.71	0.54	179.5	21.6	18.5	1.72	2.30	8.32	9.70
Pebax-2533/SSMMP 20 wt%	1.25	0.71	0.54	188.9	22.0	25.3	1.76	2.30	8.59	7.47
Pebax-2533/SSMMP@PIM-1 0.5 wt%	1.23	0.74	0.57	145.5	18.8	13.8	1.67	2.15	7.74	10.58
Pebax-2533/SSMMP@PIM-1 10 wt%	1.26	0.72	0.58	181.8	23.3	20.3	1.75	2.19	7.82	8.97
Pebax-2533/SSMMP@PIM-1 20 wt%	1.28	0.74	0.60	230.2	26.8	23.9	1.72	2.12	8.60	9.62
Pebax-2533/SSMMP-NH_2 0.5 wt%	1.14	0.71	0.58	154.5	18.0	13.2	1.60	1.97	8.58	11.70
Pebax-2533/SSMMP-NH_2 10 wt%	1.13	0.70	0.55	240.7	24.2	14.2	1.61	2.04	9.96	17.00
Pebax-2533/SSMMP-NH_2 20 wt%	1.06	0.68	0.54	301.1	24.4	16.6	1.56	1.98	12.32	18.11
Pebax-2533/SSMMP-NH_2@PIM-1 0.5 wt%	1.26	0.78	0.62	147.1	17.3	13.2	1.62	2.05	8.50	11.12
Pebax-2533/SSMMP-NH_2@PIM-1 10 wt%	1.28	0.80	0.63	228.6	23.2	16.7	1.60	2.05	9.88	13.72
Pebax-2533/SSMMP-NH_2@PIM-1 20wt%	1.41	0.87	0.65	306.1	30.8	20.3	1.61	2.18	9.92	15.10

[a] Diffusivity coefficient [cm^2/s]×10^6.

[b] Solubility coefficient [cm^3 (STP)/cm^3 cmHg]×10^4.

[c] Values from Bernardo et al.[1] (1 bar, 25°C).

[d] Values from Hassanzadeh et al.[2] (2 bar, 30°C) using PlotDigitizer free online app.

Table S2. Comparison of CO_2/N_2 and CO_2/CH_4 separation performance of the MMMs produced in this study with those in literature.

Polymer	Filler	Loading (wt%)	Conditions			P CO_2 (Barrer)	Selectivity		Ref
			P (bar)	T (°C)	Type		CO_2/CH_4	CO_2/N_2	
Pebax-2533	ZIF-8-PEI@IL	15	2	24	Ideal	285	25	76	[3]
Pebax-2533	Sorbitol	20	10	30	Ideal	462.1	12.87	47.15	[2]
Pebax-2533	ZIF-7-CH3OH	10	4.5	25	Ideal	562	-	19	[4]
Pebax-2533	ZIF-11	70	2	20	Ideal	402.89	12.49	-	[5]
Pebax-1657	ZIF-8@PIM-1	20	3	35	Ideal	105	18.8	45.6	[6]
Pebax-2533	One-pot synthesized ZIF-8	8	10	25	Ideal	184.2	27.3	50.7	[7]
Pebax-2533	UiO-67-bpdc0	2	10	30	Ideal	761.1	17.1	-	[8]
Pebax-2533	Zn/Ni-ZIF-8	10	5	25	Ideal	408	-	51.8	[9]
Pebax-2533	NPC	5	2	25	Ideal	553	-	56.4	[10]
Pebax-2533	POSS	0.1	4	25	Ideal	194.35	5.67	27.07	[11]
Pebax-2533	SSMMP-NH_2@PIM-1	20	4	25	Ideal	431.1	16.1	32.9	This work
Pebax-2533	SSMMP-NH_2@PIM-1	20	7	25	Ideal	446.5	20.8	36.3	This work
Pebax-2533	SSMMP-NH_2@PIM-1	20	10	25	Ideal	501.7	23.2	42.9	This work

Table S3. Pure gas permeances of PSF-PDMS TFC

Membranes	Permeance (GPU)			Ideal Selectivity		Ref
	CO_2	CH_4	N_2	CO_2/CH_4	CO_2/N_2	
PSF-PDMS	3655 ± 128	929 ± 48	651 ± 42	3.9	5.6	This work
PAN-PDMS	4050	-	-	-	9	[12]
PAN-BF-PDMS [a]	3704 ± 340	-	370 ± 20	-	10.0	[13]

[a] Pre-wetted condition and Sylgard 184 as PDMS

Note S1. Estimating the thickness of the selective layer and protective layer

Utilizing the resistance-in-series model [14,15], the thicknesses of the TFC layers were determined using the permeability values of MMM and pure PDMS, as well as the permeance values of the PSF porous substrate. Penetrations of the layers into the porous structure were not considered for the calculation.

The thickness of the selective layer based on Pebax 2533/SSMMP-NH_2@PIM-1 20 wt% was initially determined as follows:

$$J_{MMM-PSF} = \left(\frac{L_{MMM}}{P_{MMM}} + \frac{L_{PSF}}{P_{PSF}}\right)^{-1}$$

$$J_{MMM-PSF} = \left(\frac{L_{MMM}}{P_{MMM}} + J_{PSF}{}^{-1}\right)^{-1}$$

$$621 = \left(\frac{L_{MMM}}{431.1} + 9450^{-1}\right)^{-1}$$

$$L_{MMM} = 0.65\ \mu m$$

Where $J_{MMM-PSF}$ is the CO_2 permeance of the TFC produced only with the selective layer (621 GPU, according to experiment), L_{MMM} is the thickness of the selective layer, P_{MMM} is the CO_2 permeability of the selective layer (431.1 Barrer), and L_{PSF}, P_{PSF}, and J_{PSF} are the thickness, CO_2 permeability and CO_2 permeance of the PSF porous substrate (9450 GPU, according to experiment).

Subsequently, utilizing the thickness identified for the selective layer, it is feasible to ascertain the thickness of the protective layer by employing the CO_2 permeance of the TFC produced with all layers:

$$J_{PDMS-MMM-PSF} = \left(\frac{L_{PDMS}}{P_{PDMS}} + \frac{L_{MMM}}{P_{MMM}} + \frac{L_{PSF}}{P_{PSF}}\right)^{-1}$$

$$J_{PDMS-MMM-PSF} = \left(\frac{L_{PDMS}}{P_{PDMS}} + \frac{L_{MMM}}{P_{MMM}} + J_{PSF}{}^{-1}\right)^{-1}$$

$$575 = \left(\frac{L_{PDMS}}{3970} + \frac{0.65}{431.1} + 9450^{-1}\right)^{-1}$$

$$L_{PDMS} = 0.51\ \mu m$$

Where $J_{PDMS-MMM-PSF}$ is the CO_2 permeance of the TFC presented in Table 7 (575 GPU), L_{PDMS} is the thickness of the protective layer, and P_{PDMS} is the CO_2 permeability of the protective layer (3970 Barrer, obtained at work [16]).

In this manner, it was feasible to determine the thickness of the selective layer to be 0.65 μm and the thickness of the protective layer to be 0.51 μm.

References

(1) Bernardo, P.; Jansen, J. C.; Bazzarelli, F.; Tasselli, F.; Fuoco, A.; Friess, K.; Izák, P.; Jarmarová, V.; Kačírková, M.; Clarizia, G. Gas Transport Properties of Pebax®/Room Temperature Ionic Liquid Gel Membranes. In *Separation and Purification Technology*; 2012; Vol. 97, pp 73–82. https://doi.org/10.1016/j.seppur.2012.02.041.

(2) Hassanzadeh, H.; Abedini, R.; Ghorbani, M. CO2 Separation over N2 and CH4 Light Gases in Sorbitol-Modified Poly(Ether-Block-Amide) (Pebax 2533) Membrane. *Ind Eng Chem Res* 2022, *61* (36), 13669–13682. https://doi.org/10.1021/acs.iecr.2c02760.

(3) Li, G.; Kujawski, W.; Tonkonogovas, A.; Knozowska, K.; Kujawa, J.; Olewnik-Kruszkowska, E.; Pedišius, N.; Stankevičius, A. Evaluation of CO2 Separation Performance with Enhanced Features of Materials – Pebax® 2533 Mixed Matrix Membranes Containing ZIF-8-PEI@[P(3)HIm][Tf2N]. *Chemical Engineering Research and Design* 2022, *181*, 195–208. https://doi.org/10.1016/j.cherd.2022.03.023.

(4) Gao, J.; Mao, H.; Jin, H.; Chen, C.; Feldhoff, A.; Li, Y. Functionalized ZIF-7/Pebax® 2533 Mixed Matrix Membranes for CO2/N2 Separation. *Microporous and Mesoporous Materials* 2020, *297*, 110030. https://doi.org/10.1016/J.MICROMESO.2020.110030.

(5) Ehsani, A.; Pakizeh, M. Synthesis, Characterization and Gas Permeation Study of ZIF-11/Pebax® 2533 Mixed Matrix Membranes. *J Taiwan Inst Chem Eng* 2016, *66*, 414–423. https://doi.org/10.1016/j.jtice.2016.07.005.

(6) Kang, C.; Moon, Y.; Kim, J. E.; Kim, H.; Cho, J.; Hong, J.; Park, J.; Kim, B. G. Enhanced CO□ Separation Performance of Mixed-Matrix Membranes through PIM-1 Based Surface Engineering Using Non-Solvent Induced Surface Deposition. *J Memb Sci* 2025, *721*. https://doi.org/10.1016/j.memsci.2025.123838.

(7) Maleh, M. S.; Raisi, A. Preparation of High Performance Mixed Matrix Membranes by One-Pot Synthesis of ZIF-8 Nanoparticles into Pebax-2533 for CO2 Separation. *Chemical Engineering Research and Design* 2022, *186*, 266–275. https://doi.org/10.1016/J.CHERD.2022.08.009.

(8) Kavianpour, M. A.; Abedini, R. Enhanced CO2 Separation Performance of Pebax®2533 Mixed Matrix Membrane Incorporated by Synthesized Mixed-Ligand UiO-67. *Chin J Chem Eng* 2025. https://doi.org/https://doi.org/10.1016/j.cjche.2025.05.002.

(9) Zhang, X.; Zhang, T.; Wang, Y.; Li, J.; Liu, C.; Li, N.; Liao, J. Mixed-Matrix Membranes Based on Zn/Ni-ZIF-8-PEBA for High Performance CO2 Separation. *J Memb Sci* 2018, *560*, 38–46. https://doi.org/10.1016/j.memsci.2018.05.004.

(10) Wang, Y.; Ma, Z.; Zhang, X.; Li, J.; Zhou, Y.; Jin, Z.; Li, N. Mixed-Matrix Membranes Consisting of Pebax and Novel Nitrogen-Doped Porous Carbons for CO2 Separation. *J Memb Sci* 2022, *644*, 120182. https://doi.org/10.1016/j.memsci.2021.120182.

(11) Khalifeh, M.; Khosravi, A.; Hashemifard, S. A. Fabrication and Characterization of Novel Pebax2533/POSS-FS Nanocomposite Membranes for CO2 Removal. *Chemical Engineering Research and Design* 2024, *201*, 551–560. https://doi.org/10.1016/j.cherd.2023.12.017.

(12) Scofield, J. M. P.; Gurr, P. A.; Kim, J.; Fu, Q.; Kentish, S. E.; Qiao, G. G. Development of Novel Fluorinated Additives for High Performance CO2 Separation Thin-Film Composite Membranes. *J Memb Sci* 2016, *499*, 191–200. https://doi.org/10.1016/j.memsci.2015.10.035.

(13) Li, P.; Chen, H. Z.; Chung, T.-S. The Effects of Substrate Characteristics and Pre-Wetting Agents on PAN–PDMS Composite Hollow Fiber Membranes for CO2/N2 and O2/N2 Separation. *J Memb Sci* 2013, *434*, 18–25. https://doi.org/10.1016/j.memsci.2013.01.042.

(14) Liang, C. Z.; Chung, T.-S.; Lai, J.-Y. A Review of Polymeric Composite Membranes for Gas Separation and Energy Production. *Prog Polym Sci* 2019, *97*, 101141. https://doi.org/10.1016/j.progpolymsci.2019.06.001.

(15) Zhang, X.; He, M.; Chen, Y.; Jia, W.; Ren, M.; Chen, C.; Chen, J.; Liu, Z.; Zhao, J.; Jin, W. Ultra-Permeable Poly(Ethylene Oxide)-Based Thin-Film Composite Membranes for High-Efficiency Carbon Capture. *J Memb Sci* 2025, 124587. https://doi.org/10.1016/j.memsci.2025.124587.

(16) Berean, K.; Ou, J. Z.; Nour, M.; Latham, K.; McSweeney, C.; Paull, D.; Halim, A.; Kentish, S.; Doherty, C. M.; Hill, A. J.; Kalantar-Zadeh, K. The Effect of Crosslinking Temperature on the Permeability of PDMS Membranes: Evidence of Extraordinary CO2 and CH4 Gas Permeation. *Sep Purif Technol* 2014, *122*, 96–104. https://doi.org/10.1016/j.seppur.2013.11.006.
